# Selection in the Finnhorse, a native all‐around horse breed

**DOI:** 10.1111/jbg.12524

**Published:** 2020-11-23

**Authors:** Laura Kvist, Johanna Honka, Markku Niskanen, Oona Liedes, Jouni Aspi

**Affiliations:** ^1^ Department of Ecology and Genetics University of Oulu Oulu Finland; ^2^ Research Unit of History, Culture and Communications University of Oulu Oulu Finland

**Keywords:** coat colour, locomotion, outlier SNP, size at withers, whole‐genome analysis

## Abstract

Selection by breeders modifies the morphology, behaviour and performance of domesticated species. Here, we examined signs of selection in Finnhorse, the only native horse breed in Finland. We first searched divergent genomic regions between Finnhorses and other breeds, as well as between different breeding sections of the Finnhorse with data from Illumina Equine SNP70 BeadChip, and then studied several of the detected regions in more detail. We found altogether 35 common outlier SNPs between Finnhorses and other breeds using two different selection tests. Many of the SNPs were located close to genes affecting coat colour, performance, size, sugar metabolism, immune response and olfaction. We selected genes affecting coat colour (*KIT*, *MITF*, *PMEL*), performance (*MSTN*) and locomotion (*DMRT3*) for a more detailed examination. In addition, we looked for, and found, associations with height at withers and SNPs located close to gene *LCORL*. Among the four breeding sections of Finnhorses (harness trotters, riding horses, draught horses and pony‐sized horses), a single SNP located close to the *DMRT3* gene was significantly differentiated and only between harness trotters and pony‐sized horses.

## 1 INTRODUCTION

Domestication fundamentally changes the behaviour, morphology, physiology and performance of animal species through selective breeding—breeders’ decisions of which individuals will be allowed to reproduce. This artificial selection is often directional, decreasing the amount of genetic diversity within breeds. Genetic diversity can decrease also because of fixation of breed‐specific traits through inbreeding, genetic drift and founder effect (Frantz et al., 2020; Mignon‐Grasteau et al., 2005). On the other hand, different breeds of a given domesticated species can vary considerably due to artificial diversifying selection. Examples of large phenotypic differences between breeds and between wild and domesticated animals are the huge variations in coat colour (Cieslak et al., 2011) or body size (e.g., in dogs, *Canis lupus familiaris*; Beale & Ostrander, 2012), which are common in domesticated species, but often show limited variation within breeds. Thus, captive breeding may on the one hand decrease overall phenotypic and genetic variation and on the other hand increase it for certain traits.

Horses (*Equus caballus*) have been selectively bred for centuries, especially for transportation, agriculture and warfare, nowadays mainly for leisure and sports activities. Schubert et al. (2014) identified altogether 125 genomic regions, which have potentially been affected by the domestication process in the horse. They classified these regions into two groups, of which the first consisted of genes involved in muscular and limb development, articular junctions and the cardiac system (physiological adaptations), and the second consisted of genes with cognitive functions (tameness). Several of the genomic changes accompanying domestication have indeed been related to forelimb robustness, performance, gaits, cognitive skills and behaviour, but also to coat colour, many of which support the “neural crest hypothesis for domestication” (Librado et al., 2017; Ludwig et al., 2009; Petersen, Mickelson, Rendahl, et al., 2013); that is, development of number of these traits is linked to neural crest cells (Wilkins et al., 2014). Much research has recently focused on these traits in horses, especially on genes affecting colour, gait patterns and performance (reviewed, e.g., in Raudsepp et al., 2019). Connected with the “neural crest hypothesis of domestication,” mutations in genes affecting tissues originating from the neural crest cells have been found also to cause several diseases. Such diseases are, for example, the lethal white syndrome and stationary night blindness in horses (Metallinos et al., 1998; Raudsepp et al., 2019), which affect also coat colour. In general, genetic load in horses has increased and diversity decreased especially during the last couple of hundreds of years, due to modern breeding practices (Fages et al., 2019; Raudsepp et al., 2019).

Finnhorse as a breed was officially founded with the establishment of the studbook in 1907. We refer native horses of Finland predating this year as Finnish horses. These Finnish horses formed three main types in the early 20th century: (a) heavy horses used in agriculture; (b) lightly built and “leggy” horses used in harness racing; and (c) relatively light, pony‐sized horses, in addition to local landraces. The breed was strongly selected for colour and size in the early years. Only horses representing the heavy type were accepted in the studbook between 1907 and 1924, because agriculture and forestry required draft‐type horses. However, as trotting speed was desired in addition to weight pulling, most stallions represented a light‐draft phenotype. Accepted stallions had to be at least 148 cm tall at the withers (Ojala et al., 2007; Peltonen, 2014). There was also intense selection for colour, with chestnuts strongly favoured, and “foreign” colours (white, grey, palomino and piebald) not accepted. In 1871, only about 23% of the Finnish horses were chestnuts, contrary to 1920, during which over 85% of the Finnhorses accepted to the studbook were chestnuts (Perttunen, 2007).

Due to the Finnish military's need for riding horses, the Finnhorse studbook was divided into two sections in 1924, heavier draft‐type horses and light‐type riding horses. Light horses with phenotypic traits of warmblood riding horses (e.g., an open throatlatch, a longer neck), excluded from the studbook since 1907, were thus now accepted. However, favouring of draft‐type phenotypes since 1907 had already largely depleted suitable breeding material for light horses (Ojala et al., 2007), and in the late 1940s, it was actually noted that the overall suitability of the Finnhorse for riding had deteriorated over time (Ojala, 1997, 2007). The light‐type horse section that included also Finnhorses competing in harness trotting was renamed as the universal horse section in 1935 and was replaced by a trotting horse section in 1965 (Ojala, 1997; Ojala et al., 2007).

Since 1971, Finnhorses have been bred in four breeding sections: harness trotters, riding horses, pony‐sized horses and draught horses. Horses of each breeding section need to fill section‐specific criteria for acceptance to a breeding section. This has brought additional selection for traits specific to these breeding sections (e.g., performance, gait, size and conformation; Ojala, 2007). Registered Finnhorses form a common gene pool, from which new horses can be accepted to a breeding section after individual evaluation. Thus, a given horse can belong to several breeding sections and offspring do not automatically belong to the breeding section(s) of their parents (The Finnish Trotting and Breeding Association, 2017). Most Finnhorses are not evaluated and do not belong to any of the breeding sections. This likely leads to a quite weak effect of selection within the breeding sections. The studbook has been closed for 110 years and provides pedigrees and much of other information of all Finnhorses (The Finnish Trotting and Breeding Association, 2017).

Recent analyses of genomic SNPs and mitochondrial DNA in Finnhorses showed that mitochondrial genetic diversity and female effective populations sizes are high (Kvist et al., 2019), which is common for all horse breeds (e.g., Jansen et al., 2002; Raudsepp et al., 2019). On the other hand, effective population size (N_e_) from the nuclear SNP data was just about 50 for each of the breeding sections and for the breed as a whole (Kvist et al., 2019). For comparison, N_e_ calculated from genome‐wide SNP data from close to 40 breeds varied between 143 and 751 (Petersen, Mickelson, Cothran, et al., 2013), and in several native breeds with small populations, N_e_ was considerably smaller (e.g., 41 in South Korean Jeju horses; Do et al., 2014, 39 in Italian Bardigiano horses; Ablondi et al., 2020, and 40–85 in several native Polish breeds; Jasielczuk et al., 2020). Based on inbreeding coefficients and number of runs of homozygosity (ROHs), inbreeding has not been very high compared with other breeds, but ROHs were on average longer than in several other breeds (Kvist et al., 2019). There are not many studies of specific phenotypic or genotypic traits in Finnhorses. However, a few reports based on pedigree analyses and information from studbook have shown that size is positively correlated with racing performance and has a high heritability, whereas correlation of body conformation with racing traits is low and has a low heritability (Suontama et al., 2009, 2013). Of the specific genotyped traits, the *DMRT3* (*Doublesex And Mab‐3 Related Transcription Factor* 3) gene alleles have been found to affect speed in trotting horses and quality of gaits in riding horses (Jäderkvist et al., 2015).

Here, our aim was to study signs of selection in Finnhorses to obtain more information about genes and genomic regions that have been affected by human‐mediated selection. We explored whether there are sites of genomic differentiation between Finnhorses and other breeds in general by first screening for candidate loci under selection by comparing SNP chip data of Finnhorses with other breeds. We also searched for signs of selection between the breeding sections (harness trotters, riding horses, pony‐sized horses and draught horses) of the Finnhorse. Based on the results of the screenings, we then studied a selected set of loci previously connected to coat colour, performance, locomotion and height at withers to identify candidate loci for a set of specific traits.

## 2 MATERIALS AND METHODS

### 2.1 Samples, questionnaires and SNP data

We analysed altogether 985 horse samples that were previously included in the study by Kvist et al., (2019). These samples included 846 registered Finnhorses of which 205 were registered in specific breeding sections. Of the horses registered in breeding sections, 67 were harness trotters, 79 riding horses, 30 draught horses and 51 pony‐sized horses. Altogether, 16 of these horses were concurrently registered in two breeding sections (three in both trotter and riding, three in riding and draught, five in riding and pony‐sized, one in draught and pony‐sized and four in trotter and draught). Additional 139 horses of 27 other breeds (and one of unknown breed from Estonia), including six crossbreds, were sampled (Kvist et al., 2019; Table 1). Samples were obtained either from horse owners (hair samples from mane or tail, *N* = 954) or from an equine veterinary hospital (blood samples, with the permission from the owners, *N* = 31). DNA was extracted from these samples as in Kvist et al. (2019).

**TABLE 1:** Breeds and numbers of horses included in the study

Breed	*N*	*N*, Equine SNP70
American Quarter	1	1
Crossbred pony	3	
Crossbred coldblood	1	
Estonian horse	2	1
Estonian riding pony	1	
Estonian Sport Horse	2	
Finnhorse	846	60
Finnish Warmblood (FWB)	7	1
Friesian horse	1	
German Riding Pony	1	
Gotland Russ Pony	3	1
Hanoverian horse	2	
Icelandic horse	1	
Irish Cob	2	1
Latvian Sport Horse	2	
Norwegian Fjord	1	1
Oldenburg horse	1	
Pura Raza Española (PRE)	23	
Puro Sangue Lusitano (PSL) (including mixed PRE/PSL)	3	
Shetland pony	39	2
Sorraia Pony	1	
Tori horse	1	
Royal Dutch Sport Horses (KWPN)	2	1
Unknown Estonian origin	1	
Warmblood Riding horse	3	
Warmblood Trotters	30	2
Welsh Mountain Pony	3	1
Yakut horse	1	
Zangersheide horse	1	

Note

*N* = number of individuals; *N*, Equine SNP70 = number of individuals genotyped with the Illumina Equine SNP70 BeadChip.

The horse owners filled a questionnaire (Figure S1), which included descriptions of the quality of different gaits (walk, trot and canter), information of the performance of the horse, height at the withers and coat colour and markings. The quality of walk was assessed by choosing between options for (a) beat, as pacy (at least sometimes) or four‐beat walk and (b) length of the step as long or short. Quality of trot was assessed by choosing (a) beat as two‐beat or pacing (at least sometimes), (b) length of the step as long or short and (c) height of the step as low or high. Quality of canter was assessed by choosing between (a) beat of the canter as three‐beat or four‐beat (at least sometimes), (b) roundness of the steps as round or sharp, (c) long‐ or short‐striding canter and (d) low‐ or high‐stepping canter. Performance of the horse was assessed by choosing between (a) sprinter, (b) medium‐distance or (c) long‐distance capacity. In addition, performance of harness trotters was qualified as record times in harness racing.

Of these samples, 60 Finnhorses (12 horses randomly sampled from each of the four breeding section and 12 horses not registered in any breeding section) and 12 randomly sampled horses from other breeds (Table 1; questionnaire data were available for all these samples as well) were genotyped with the Illumina Equine SNP70 BeadChip in Dr. Van Haeringen Laboratorium (Wageningen, the Netherlands). The chip includes 65 157 SNPs over the horse genome. Genotyping call rates averaged 0.9883, excluding three samples with call rates of 0.3550–0.4186 (two pony‐sized Finnhorses and one Finnhorse, which was not registered to any breeding section), which were not included in further analyses. After pruning for minor allele frequencies (*maf* = 0.05) and linkage equilibrium (with a sliding window of 50 SNPs, shifting the window 5 SNPs forward and removing SNPs with *r* ^2^ > .5; ‐‐*maf* 0.05 ‐*indep*‐*pairwise* 50 5 0.5) with plink 1.9 (Chang et al., 2015; Purcell & Chang, 2018; Purcell et al., 2007), 37 445 SNPs remained for further analyses (see also Kvist et al., 2019).

### 2.2 Selection

We used two approaches to detect signs of selection in the horse genomes based on *F*_ST_ outliers. The first method to identify outlier SNPs was bayescan v.2.1 (Foll & Gaggiotti, 2008). This program is based on a multinomial‐Dirichlet model and searches for differences in allele frequencies between predefined populations assuming correlated allele frequencies through a common migrant gene pool. The difference in allele frequencies between the common gene pool and each population is measured by *F*_ST_ coefficients for each locus, and a posterior probability for a model including selection is calculated. Loci with high posterior probability are considered as indicative of selection, “*F*_ST_ outlier” loci. We applied the default parameters with 5,000 outputted iterations, thinning interval of 10, 20 pilot runs with a length of 5,000 and an additional burn‐in set to 50 000. We searched for outliers between the Finnhorses and the group consisting of mixed breeds based on q‐values (minimum false discovery rate at which a locus may become significant) lower than 5%.

The second *F*_ST_ outlier‐based method used for detection of selection was the hierarchical island model implemented in program arlequin v.3.5.2.2. (Excoffier & Lischer, 2010). This method simulates a null distribution and confidence intervals of the observed values and compares locus‐specific *F*_ST_ values to global *F*_ST_ value to detect outliers (Excoffier et al., 2009). The hierarchical island model should reduce the number of false positives. We executed the selection analysis using the hierarchical model with 20 000 simulations. We also searched for outliers between pairwise comparisons of the Finnhorse breeding sections the same way.

Further, we performed a gene ontology and enrichment analysis using david v.6.8 (Huang et al., 2009) for all the identified genes located close to the SNPs that showed signs for selection, combining results from both bayescan and arlequin. We used *Equus caballus* annotation file as a background and a significance threshold of *p* < .05.

### 2.3 Screening polymorphisms in candidate genes associated with specific traits

We chose six shared outlier loci from the selection test in bayescan and arlequin (see results) for further inspection. These specific genes were selected because they have been found to be under selection in other breeds and several polymorphisms are known (Raudsepp et al., 2019). Three of the outlier loci detected by comparisons between Finnhorses and other breeds were in regions, where also several genes involved in coat colour are located.

- First of the coat colour‐associated genes, *KIT*, *proto‐oncogene receptor tyrosine kinase* gene, is involved in varying white markings in the coat (sabino, tobiano, dominant white and roan) with at least 32 variable sites (e.g., Grilz‐Segera et al., 2020). We examined the most common one, sabino1 (*SB1*), which is caused by a SNP *g2350‐13 G > A* in intron 16 leading to exon splitting and lack of exon 17. The existence of this SNP was examined as in Brooks and Bailey (2005).
- The second coat colour‐associated *F*_ST_ outlier was close to gene *MITF*, coding for *melanogenesis‐associated transcription factor*, which is also associated with white coat colour variants and markings. The most common variant, the so‐called splashed white phenotype, is caused by a 11‐bp insertion in the promoter region of *MITF* (*g.20117302Tdelins11*, *SW1*; Hauswirth et al., 2012). Existence of this insertion was searched by fragment analysis of a 112‐bp amplicon produced by PCR (PCR protocol in Table S1). The PCR products were run with ABI 3730 sequencer and scored with GeneMapper v.5 (Applied Biosystems). The white patterning usually resembles as if the horse was dipped in white paint feet and head first, and this colour pattern is also often accompanied by blue eyes. For the gene variants resulting in white markings (*KIT* and *MITF*), we selected samples from Finnhorses that showed large white markings (stockings, blaze, spots in the trunk) and/or blue eyes (*N* = 59) and a control group with no white markings (*N* = 10).
- The third coat colour outlier was at the region, where a gene involved in a dilution colour called silver is located. Silver colour is connected with two strongly linked SNPs at the *PMEL* gene coding for *premelanosome protein*. This mutation fades the mane and tail of eumelanin (black pigment)‐producing horses to flaxen or silver and body colour to chocolate or reddish, but it does not have a fading effect on pheomelanin‐producing horses, that is chestnuts (Reissman et al., 2007). The SNPs are also linked with an allele 177 in a microsatellite locus *TKY284* (Brunberg et al., 2006), which we genotyped from 550 Finnhorses by fragment analysis of PCR products run on ABI 3730 sequencer and scored with genemapper v.5 (Applied Biosystems). We then examined the SNP *g1457 C > T* that changes an amino acid arginine to cysteine in exon 11 of the *PMEL* gene (Brunberg et al., 2006; Reissman et al., 2007). For this analysis, we chose samples of Finnhorses, which were registered as silver (*N* = 5), had the allele 177 in preliminary analyses with a microsatellite locus *TKY284* (*N* = 10) and/or had flaxen or faded coloured mane and tail (*N* = 17). We amplified and sequenced a 182‐bp fragment (PCR and sequencing protocols in Table S1), run the reactions on ABI 3730 automatic sequencer (Applied Biosystems) and scored alleles from the electropherograms using seqscanner v.2 (Applied Biosystems).

One of the *F*_ST_ outliers was close to the *myostatin* gene *MSTN*, which has previously been connected with racetrack performance (Hill et al., 2010; McGivney et al., 2012). Variation in the intron of this gene (*g.66493737C > T*) producing SNP genotypes TT and CT has been associated with better performance in long distances and CC genotypes with better performance in short distances (McGivney et al., 2012). We used short amplicon sequencing for detection of the SNP by amplifying and sequencing a 108‐bp fragment from all our samples (PCR and sequencing protocols in Table S1). The reactions were run and alleles scored as above for *PMEL* gene. Genotype frequencies were calculated for the four Finnhorse breeding sections and Finnhorses not included into any of the breeding sections, the mixed‐breed group, and for subtracted groups of three breeds with over 20 samples (warmblood trotters, Pura Raza Española [PRE] horses and Shetland ponies). The Hardy–Weinberg equilibrium and differences in genotype frequencies between the four Finnhorse breeding sections and horses not included in the breeding sections were tested with the *G* test. Note that here those horses, which were registered in two breeding sections, were included in both sections. Further, we tested differences in *MSTN* genotype distribution between Finnhorses classified into different endurance classes (classification into sprinters, medium‐ or long‐distance runners by owners) and used for different purposes (riding, trotting, combined driving, draught, eventing) using the *G* test. Additionally, we tested whether there were differences in records of harness racing competitions between the genotypes using the *t* test. Here, we included record times only of horses aged 6 years or more.

We used short amplicon sequencing also for obtaining allele frequencies for the “gait‐keeper” gene, *doublesex and mab‐3‐related transcription factor 3* (*DMRT3*). A nonsense mutation in this gene has been shown to affect the capability of horses to pace and amble (*DMRT3_Ser301STOP*; Andersson et al., 2012). The “gaited” genotype (AA) has been connected with good performance in Finnhorse trotters and the heterozygote genotype (CA) and homozygote (CC) with good quality gaits in riding horses (Jäderkvist et al., 2015). We amplified and sequenced a 100‐bp fragment (PCR and sequencing protocols in Table S1), scored the alleles, calculated genotype frequencies and tested Hardy–Weinberg equilibrium as described above for the *MSTN* gene. In addition, we tested for differences in *DMRT3* genotypes in horses classified according to their gait quality, with the criteria explained above, using the *G* test.

One of the detected outliers was located close to gene *LCORL* that codes for a *ligand‐dependent nuclear receptor corepressor like protein*. This is one of the major loci suggested to affect height at the withers (Makvandi‐Nejad et al., 2012; Signer‐Hasler et al., 2012). To look more into this, we performed a genome‐wide association analysis (GWAS) with the SNP data and height at withers in plink.1.9 (‐‐*assoc*).

## 3 RESULTS

### 3.1 Outliers

**FIGURE 1:**
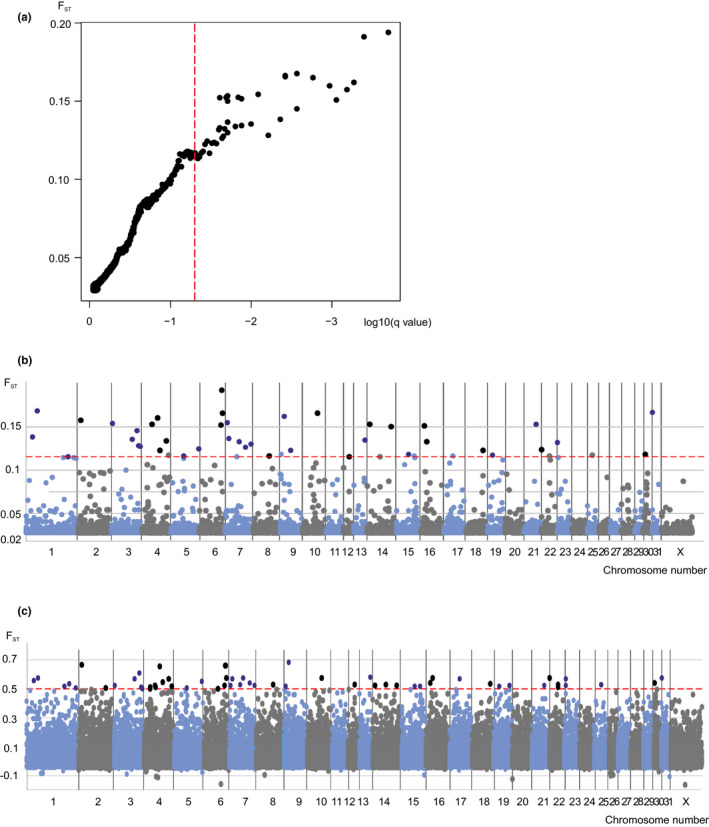
Results from the selection analysis. (a) *F*_ST_ values of all loci plotted against log_10_(*q*‐value) from bayescan. The dashed vertical line shows the log_10_(*q*‐value) equalling *q* = 0.05. (b) *F*_ST_ values from bayescan plotted along the 31 autosomal equine chromosomes and X chromosome. Dashed red horizontal line = smallest *F*_ST_ (0.1133) of the loci with log10(*q*‐value) equalling *q* = 0.05, shaded colours above the line = loci with log10(*q*‐value) above *q* = 0.05. (c) *F*_ST_ values from arlequin plotted along the 31 autosomal equine chromosomes and X chromosome. *F*_ST_ = 0.5 is shown by a dashed red horizontal line and shaded colours above the line = loci with *F*_ST_ > 0.5 [Colour figure can be viewed at wileyonlinelibrary.com]

**FIGURE 2:**
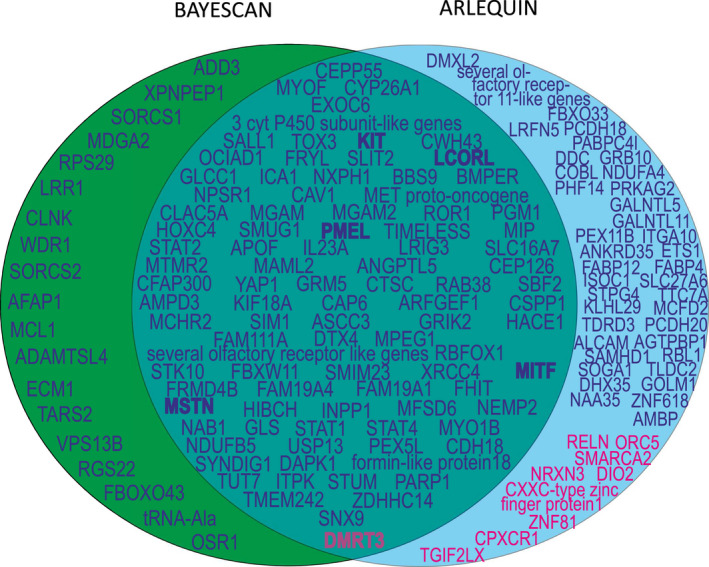
A Venn diagram showing the genes located at the regions of outlier SNPs. Green background shows the results from bayescan, blue background the results from arlequin and intersection the genes located at the regions of outlier SNPs detected by both methods. Blue text denotes the results from comparisons between Finnhorses and other breeds, red the comparison between pony‐sized Finnhorses and harness trotters and bold the candidate genes associated with specific traits examined in more detail. Full names of the genes are in Table S2 [Colour figure can be viewed at wileyonlinelibrary.com]

bayescan detected altogether 42 and arlequin 55 outlier loci with *F*_ST_ values ranging 0.11326–0.19392 and 0.5000–0.6802, respectively, in comparisons between Finnhorses and other breeds (Figures 1 and 2; Table S2). Mean *F*_ST_ over all loci was 0.0025 (*SD* = 0.0006) in bayescan and 0.0370 (*SD* = 0.0825) in arlequin. The two different methods detected altogether 35 common loci. The common outlier loci were found in chromosomes 1 (1 SNPs), 2 (1 SNP), 3 (3 SNPs), 4 (4 SNPs), 5 (1 SNPs), 6 (4 SNPs), 7 (5 SNPs), 8 (1 SNP), 9 (1 SNPs), 10 (1 SNP), 12 (1 SNP), 13 (1 SNP), 14 (2 SNPs), 16 (2 SNPs), 18 (1 SNP), 19 (1 SNP), 21 (1 SNP), 22 (1 SNP), 23 (1 SNP), 30, (1 SNP) and 31 (1 SNP). Four of those loci were in regions where also genes involved in coat colour can be found (chromosome 3, *KIT*, *proto‐oncogene receptor tyrosine kinase* that is involved in white markings in the coat; chromosome 6, *PMEL*, *premelanosome protein* that is known to be involved in silver coat colour; chromosome 10, *MCHR2*, *melanin concentrating hormone receptor 2* that is a possible candidate for colour; and chromosome 16, *MITF*, *melanogenesis‐associated transcription factor* that is associated with white coat colour variants and markings, “splashed white”). One locus in chromosome 3 was close to *LCORL*, *ligand‐dependent nuclear receptor corepressor like*, a gene that has been connected with body size, and one locus in chromosome 18 was close to *MSTN*, *myostatin* gene that has been connected with performance (e.g., sprinter or long‐distance runner types). Several loci are located close to genes, which regulate sugar metabolism (e.g., chromosome 1, *SORCS1*, *Sortilin Related VPS10 Domain Containing Receptor 1*; chromosome 4, *ICA1*, *islet cell autoantigen 1*, *CAV1*, *caveolin 1*, *MGAM*, *maltase‐glucoamylase* and *MGAM2*, *maltase‐glucoamylase 2*). In addition, several SNPs were found close to genes involved in immune response (e.g., chromosome 3, *CLNK*, *cytokine‐dependent haematopoietic cell linker 4*; chromosome 4, *NPSR1, neuropeptide S receptor 1*, *CLEC5A*, C*‐type lectin domain containing 5A*; chromosome 6; *IL23A*, *interleukin‐23 alpha subunit p19*; chromosome 14, *FBXW11*, *F‐box and WD repeat domain containing 11*; and chromosome 18, *STAT1* and *STAT4*, *signal transducer and activator of transcription 1 and 4*). Two SNPs were in regions holding many genes of the olfactory receptor family in chromosomes 1 and 12. In comparisons between breeding sections of the Finnhorse, only one outlier was detected by bayescan and five by arlequin in comparisons between harness trotters and pony‐sized horses. The SNP BIEC_2_620406 located in chromosome 23, about 500 kb upstream of the *DMRT3* gene, *doublesex and mab‐3‐related transcription factor 3* (Table S2), was found by both analyses (bayescan *p* < .022). A Venn diagram of genes located at the regions of the outlier SNPs detected with BAYESCAN and ARLEQUIN and an intersection detected by both approaches is shown in Figure 2.

Gene ontology and enrichment analysis by david found that the most important functional annotation clusters were involved in (a) signal transduction and transcriptional regulation, (b) endomembrane systems, (c) apoptosis, (d) cell cortex, (e) cellular responses (to a variety of stimuli) and (f) immunity (Table S3 and Figure S2).

### 3.2 Polymorphisms in candidate genes associated with specific traits

#### 3.2.1 Coat colour

We found seven Finnhorses carrying the splashed white (*SW1*) allele as heterozygotes and no sabino1 (*SB1*) carriers out of the 59 candidates having white markings and/or blue eyes. None of the control horses carried *SW1* or *SB1* genotypes. Studying the pedigrees of *SW1* horses revealed that four of these horses were offspring of the same stallion, whose half‐sister was also tested positive for the *SW1* mutation. Of the other two horses, one was offspring of another stallion, also registered as carrying *SW1*, and one did not have any ancestors registered as *SW1*.

Fourteen of the 32 suspected carriers of the silver mutation were heterozygotes for the mutation, all the rest were homozygous for the wild allele. One of the homozygotes for the wild allele carried the 177 allele of microsatellite *TKY284* linked with the silver mutation, two of the heterozygotes did not carry the 177 allele and three failed to amplify. Seven of the fourteen heterozygotes shared a same silver stud in their pedigrees and two shared another silver stud, the remaining five did not share any recent ancestors.

#### 3.2.2 Performance and gaits

The *MSTN* genotypes showed no differences between Finnhorse breeding sections nor in horses not registered in any of the breeding sections (*G* test values 0.665–5.109, all *p*‐values >.070; Table 2). However, the difference between Finnhorses and mixed‐breed group was highly significant (*G* = 32.773, *p* < .001), there were in general much more TT homozygotes (long‐distance runners) and less CC homozygotes (sprinters) in Finnhorses than in other breeds. However, also warmblood trotters and PRE‐horses had more TT homozygotes and fewer CC homozygotes than the other breeds we studied (Table 2). We found no effect of the *MSTN* genotype on descriptions of the horses by their owners as sprinters, medium‐ or long‐distance horses (*N* = 772, *G* = 3.605, *df* = 2, *p* = .462), nor on the usage of horses (as riding, trotting, combined driving, draught, eventing; *N* = 768, *G* = 8.557, *df* = 2, *p* = .381). For trotters, there was no effect of the *MSTN* genotype on the records of the horses (car start: *N* = 76, *t* = 0.453, *p* = .652 [two‐tailed], rolling start: *N* = 99, *t* = 0.695, *p* = .488). All groups were in Hardy–Weinberg equilibrium, except the mixed‐breed group (*G* = 10.004, *df* = 2, *p* = .007).

**TABLE 2:** *MSTN* and *DMRT3* genotype frequencies in the four breeding sections, combined mixed breeds and of three breeds with more than 26 samples

	Frequency								
	Riding	Trotter	Draught	Pony‐sized	Non‐brsec	Mixed breeds	Wbl trotter	PRE	Shetland pony
MSTN	*N* = 78	*N* = 66	*N* = 29	*N* = 51	*N* = 640	*N* = 139	*N* = 30	*N* = 26	*N* = 38
TT	0.731	0.788	0.828	0.800	0.844	0.691	1.000	0.962	0.289
CT	0.256	0.212	0.103	0.160	0.147	0.194	0.000	0.038	0.447
CC	0.013	0.000	0.069	0.040	0.009	0.115	0.000	0.000	0.263
DMRT3	*N* = 78	*N* = 66	*N* = 29	*N* = 51	*N* = 641	*N* = 139	*N* = 30	*N* = 26	*N* = 38
AA	0.115	0.197	0.345	0.118	0.298	0.216	0.967	0.000	0.000
CA	0.539	0.667	0.483	0.510	0.462	0.036	0.033	0.038	0.000
CC	0.346	0.136	0.172	0.372	0.240	0.748	0.000	0.962	1.000

Note

Non‐brsc = Finnhorses not included to any breeding section, Wbl trotter = Warmblood trotters, PRE = 23 samples from Pura Raza Espanola and 3 from Puro Sangue Lusitano or mixed Pura Raza Espanola/Puro Sangue Lusitano, *N* = sample size. *MSTN* genotypes: TT = “long distance,” CT = “medium distance,” CC = “sprinter.” *DMRT3* genotypes: AA = “gaited,” good for trotters, CA and CC = “non‐gaited,” good for riding.

Finnhorses differed from the mixed‐breed group in the frequency of the *DMRT3* genotypes, there were significantly fewer CC homozygotes and more CA heterozygotes (both associated with good gaits for riding horses) than expected (*G* = 164.501, *df* = 2, *p* = .000; Table 2). There were no CC homozygotes in our sample of warmblood trotters. There were also differences between the Finnhorse breeding sections (riding horses vs. horses not in the studbook, *G* = 13.953, *df* = 2, *p* = .0009; trotters vs. horses not in the studbook, *G* = 10.195, *df* = 2, *p* = .006; pony‐sized horses vs. horses not in the studbook, *G* = 9.818, *df* = 2, *p* = .007; riding horses vs. trotters, *G* = 9.017, *df* = 2, *p* = .011; riding horses vs. draught horses, *G* = 7.786, *df* = 2, *p* = .0210; trotters vs. pony‐sized horses, *G* = 8.832, *df* = 2, *p* = .012; and draught vs. pony‐sized horses, *G* = 7.019, *df* = 2, *p* = .030), but only the difference between riding horses versus horses not in the studbook remained significant after the Bonferroni correction, riding horses having more of the CC homozygotes and less of the AA homozygotes (the “gaited” genotype). All groups were in Hardy–Weinberg equilibrium, except the mixed‐breed group (*G* = 58.800, *df* = 2, *p* < .001).

**FIGURE 3:**
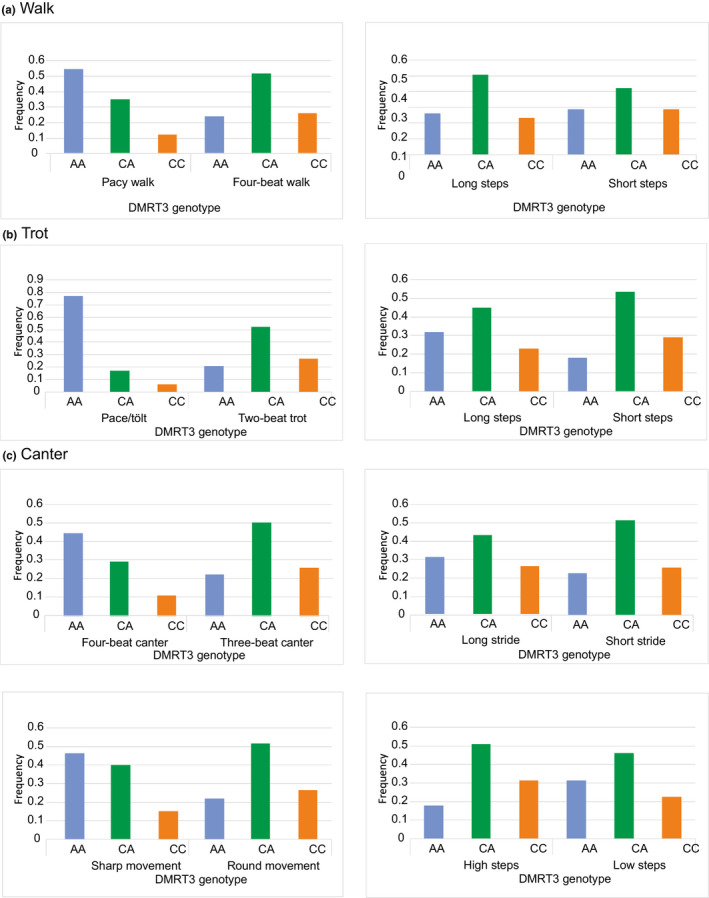
Frequencies of *DMRT3* genotypes classified according to quality of gaits among Finnhorses in (a) walk, (b) trot and (c) canter [Colour figure can be viewed at wileyonlinelibrary.com]

Finnhorses with four‐beat walk had less *DMRT3* AA homozygotes and more CC homozygotes than horses showing pacy walk (*G* = 33.819, *df* = 2, *p* < .001; Figure 3a). There was no influence of the *DMRT3* genotype to length of the steps (*G* = 4.877, *df* = 2, *p* = .087; Figure 3a). Finnhorses with two‐beat trot had less AA homozygotes and more CC homozygotes than horses showing pace (*G* = 104.595, *df* = 2, *p* < .001; Figure 3b). Horses with short trotting step length also had less AA homozygotes and more CC homozygotes and CA heterozygotes than horses with long‐trotting steps (*G* = 15.716, *df* = 2; *p* < .001; Figure 3b). There was no effect on the height of the steps (*G* = 4.695, *df* = 2, *p* = .096; data not shown) nor on the liability of trotters to break into canter (*G* = 2.249, *df* = 2, *p* = .325; data not shown). Finnhorses with three‐beat canter and round movements had less AA homozygotes and more CC homozygotes and CA heterozygotes than horses that at least sometimes present four‐beat canter and have sharp movements (*G* = 39.654, *df* = 2, *p* < .001; Figure 3c). Horses with short‐striding and high‐stepping canter also had less AA homozygotes and more CC homozygotes and CA heterozygotes than horses with long‐striding and low‐stepping canter (*G* = 6.194, *df* = 2, *p* < .05 and *G* = 16.043, *df* = 2, *p* < .001, respectively; Figure 3c). All tests with *p*‐values below .001 remained significant also after the Bonferroni correction.

#### 3.2.3 Height at withers

One SNP locus (BIEC2_849774) detected by both bayescan and arlequin in comparisons between Finnhorses and other breeds was located in chromosome 3 close to (about 180 Kb upstream) *LCORL* gene that codes for a ligand‐dependent nuclear receptor corepressor like protein (Table S2a,c) and is one of the major loci suggested to affect height at the withers (Makvandi‐Nejad et al., 2012; Signer‐Hasler et al., 2012). In addition to this SNP identified as an outlier by both bayescan and arlequin, GWAS showed the most significant result for BIEC2_808625 (*p* = 1.19 × 10^–12^), another SNP in chromosome 3 close to *LCORL* gene (about 70 Kb upstream).

## 4 DISCUSSION

Using bayescan and arlequin, we detected 42 and 55 *F*_ST_ outlier SNPs, respectively, between Finnhorses and other breeds, of which several were of special interest as they were connected to traits, which (a) were strongly selected for at the dawn of the Finnhorse breed (coat colour and size), (b) are related to the usage of the breed (performance and gaits) or (c) are associated with metabolism and immunity system. Gene ontology analysis of genes located at the proximity to the SNPs proposed to be under selection classified the genes in functional clusters including signal transduction, transcriptional regulation, cellular responses and immunity.

As noted above, several of the outliers were located close to genes affecting coat colour as expected. Most of the colour genes are involved in melanogenesis, either via synthesis of melanin, properties of melanocytes or migration of melanocytes from the neural crest (Thiruvendakan et al., 2008). However, we did not see any selection signs close to the *MC1R* gene (*Melanocortin‐1 Receptor*), which would have been expected to have arisen due to selection for chestnut colour, as this gene is responsible for production of red or black pigment. One of the detected outlier SNPs related to colour was close to *KIT*, *proto‐oncogene receptor tyrosine kinase* gene, that is crucial in melanocyte development. This gene has been connected with white coat markings. Several polymorphisms have been described in this gene, producing a variety of different white patterns in coat colouration, from totally white horses to sabinos and roans or to small patches of white in legs, face and belly (Haase et al., 2007, 2009; Hauswirth et al., 2013). In Finnhorses, roans are extremely rare, but have occasionally been detected (Perttunen, 2007). Another colour gene located close to an outlier SNP was *MITF*, *melanogenesis‐associated transcription factor* gene. This gene is as well associated with a variety of white coat colour variants and markings called splashed white. In addition, one further gene, *PAX3*, *paired box gene 3*, is connected with this type of coloration (Hauswirth et al., 2012). Based on the phenotype, it is difficult to conclude which specific variant is responsible for a particular coloration. We found evidence of only splashed white (*SW1*) causing white coat colour patches in Finnhorses, no sabinos were detected. At least three lineages were found to carry the *SW1* mutation. Previously, the rare Finnhorses with white patches were believed to be sabinos (Perttunen, 2007), but based on our results, they are more likely splashed whites.

A further colour‐related SNP outlier was located close to *PMEL*, *premelanosome protein gene*. At least two variants of this gene are linked with silver coat colour (Brunberg et al., 2006; Reissman et al., 2007). We tested 550 Finnhorses using a microsatellite linked with *PMEL* (*TKY284*; Brunberg et al., 2006) and found ten horses carrying the allele connected with silver variants. By sequencing, we confirmed fourteen silver carriers. These included all the horses registered as silver and all the horses carrying the 177 allele of the *TKY284* microsatellite, except for one horse, which carried the 177 allele but was homozygous for the wild allele at the silver locus. In addition, two of these fourteen horses did not have the 177 allele, but were found to carry the silver variant. Thus, the microsatellite allele is not perfectly linked with the silver mutation. Several lineages of Finnhorses are known to carry silver variants, and we detected the presence of the variant in seven different lineages. More of them will likely emerge with the ease of commercial testing possibilities for “colour genes.” The silver mutation is dominant; thus, heterozygotes for this mutation also express the silver phenotype except for chestnuts that lack the eumelanin pigment. All the tested horses were heterozygotes in this study. The mutation in *PMEL* gene has pleiotropic effects and is also causative of equine multiple congenital ocular anomalies (MCOA) syndrome, which is a heritable eye disorder (Andersson et al., 2013). Horses homozygote for the silver mutation also express a range of ocular defects with heterozygotes expressing much less severe symptoms (Andersson et al., 2013).

In addition to these known colour genes, one SNP outlier was located close to *MCHR2*, *melanin concentrating hormone receptor 2*. This gene has been shown to affect fish skin coloration (Takahashi et al., 2007), but the function of the protein produced by this gene is still largely unknown in mammals. In humans, it is expressed in brain and not in peripheral tissues (An et al., 2001).

Besides colour, Finnhorses were strongly selected for size at the founding of the breed. Pony‐sized horses (withers height at most 148 cm) were not accepted for breeding until the establishment of a separate breeding section for the pony‐sized Finnhorses in 1971. We detected one SNP outlier close to a transcription factor *LCORL*, *ligand‐dependent nuclear receptor corepressor like* gene. This gene has been shown to be the top locus of the four major loci, which together explained 83% of the height at withers in horses (Makvandi‐Nejad et al., 2012) and emerged as an influential locus also in a study of Franches‐Montagnes horses (Signer‐Hasler et al., 2012) and German Warmbloods (Tetens et al., 2013). Increased expression of this gene is connected to small size and decreased expression to bigger size (Metzger et al., 2013). This outlier was also strongly associated with size in our GWAS, suggesting that it indeed is connected with height at withers in Finnhorses.

Further, an SNP outlier was detected close to *MSTN*, *myostatin* gene. This gene has been connected with muscle mass, body conformation related to muscularity (e.g., skeletal sturdiness indicated by cannon bone circumference) and also performance. There are a couple of variants located in the promoter region of this gene that have been connected with heavy or light stature (Dall'Olio et al., 2014) and a variant in the first intron that has been connected with racing performance (Hill et al., 2010; McGivney et al., 2012). We sequenced the variant of the first intron and found that there were more TT homozygotes in Finnhorses compared with the mixed‐breed groups and especially so in the draught horses and in horses not registered to breeding sections. The TT genotype frequencies have been shown to be high especially in horses with greater stamina, whereas the CC homozygotes perform well in fast and short‐distance races (Bower et al., 2012; Hill et al., 2010; McGivney et al., 2012). In Finnhorses, the variants of the first intron have been found to be significantly associated with harness racing performance (Bas Con, 2017); however, this association was not found in closely related Norwegian‐Swedish Coldblooded Trotters (Velie et al., 2018) nor in Shetland ponies (Bas Con, 2017). Further, none of our outlier F_ST_s were located in the same regions detected by Velie et al. (2019) in their study of trotting racing ability that included Norwegian‐Swedish Coldblooded Trotters. We found no association of the intron variants with racing records, nor with performance type (i.e., sprinter, medium‐ or long‐distance horse) or usage of horses (riding, trotting, combined driving, draught, eventing). This might be due to long‐lasting directional selection against the CC genotypes, stemming from the use of the Finnish horses in agriculture and forestry, where greater stamina and hardiness were likely valued over sprinting performance for horses working long hours. Conformation other than that directly related to muscularity also matters in the athletic performance of horses, which likely partly explains why we found no association of the intron variants with harness racing records and performance types. After all, a TT homozygote horse with suitable proportional relationships of body and limb segments for speed is almost certainly faster than a CC homozygote horse with an unsuitable conformation for speed from purely biomechanical reasons. Unfortunately, genotypes associated with most “conformation phenotypes” (e.g., relative lengths of limb segments, neck, back, etc.) are still unknown.

The “gait‐keeper” gene *DMRT3* has been connected with harness racing performance as well. A premature stop codon in the gene allows alternate gaits, pace and ambling gaits due to its effect on the development of the locomotor network in the spinal cord. This “gaited” phenotype (genotype AA) appears in high frequency in Icelandic horses, famous for their ability to perform four or five gaits (Andersson et al., 2012), and is suggested to originate from mediaeval England (Wutke et al., 2015). It has also been suggested that the “gaited” mutation arose during or soon after the domestication of horses and was later imported to England (Staiger et al., 2017). In a survey of 141 breeds, AA genotypes were frequent in gaited breeds and breeds used for harness trotting (Promerová et al., 2014). In Finnhorses, it has already been shown that AA genotypes perform better in harness racing and CC and CA genotypes have better quality gaits for riding (Jäderkvist et al., 2015) and many individuals are known to show pace in walk or trot, at least occasionally. In Nordic coldblood trotters, AA genotypes were also shown to perform better in harness racing than CA and CC genotypes and they had higher estimated breeding values for trotting performance as well (Jäderkvist et al., 2014). We found indications of selection for this gene in comparisons between breeding sections of trotters and pony‐sized horses, higher frequencies of AA genotypes in trotters and draught horses compared with riding and pony‐sized horses and higher frequency of CC genotypes in riding and pony‐sized horses compared with trotters and draught horses. Compared with the mixed‐breed group, CA heterozygotes are more common in Finnhorse, perhaps due to the common gene pool and the “all‐around” usage of the breed as a whole. We studied the effect of the genotypes in more detail than previously using the questionnaire with detailed questions related to quality of the walk, trot and canter, and it seemed that the clearest effect is related to the rhythm (beat) of the steps in all gaits. However, there is room for errors, as the questions could not be standardized in any way.

Of the other SNP outliers detected, several were found in regions where genes regulating glucose and other carbohydrate metabolism are located, and many of those have been connected with diabetes in humans (e.g., *SORCS1*, *Sortilin Related VPS10 Domain Containing Receptor 1* in chromosome 1; *CAV1*, *caveolin 1*, *MGAM*, *maltase‐glucoamylase* and *MGAM2*, *maltase‐glucoamylase 2* in chromosome 4; Table S2). Equine metabolic syndrome (EMS) is a cluster of clinical abnormalities that predispose horses to laminitis, caused by insulin resistance and/or obesity (Geor, 2008). Many pony and native horse breeds are suggested to have high risk of EMS, likely due to their high metabolic efficiency, leading easily to obesity. Profuse ingestion of carbohydrates, especially starch from pastures, is commonly causing the acute form of laminitis (Katz & Bailey, 2012). Even though there are no studies of prevalence of laminitis in Finnhorses that we are aware of, the breed does gain weight easily and laminitis is not rare.

A common disease in Finnhorses is summer eczema or insect bite hypersensitivity (IBH), an allergic skin condition thought to be caused by biting insects, especially of the genus *Culicoides* (Hallamaa, 2009). This reaction is thought to be mediated mainly through immunoglobulin E (IgE; Wagner, 2015), produced by stimulation of T‐cell‐derived cytokines (Bos et al., 1992). We found *F*_ST_ outliers in regions close to genes associated with immune system and IgE, for example *STAT1* and *STAT2* (*signal transducer and activator of transcription*) genes, *IL23A* (*interleukin 23, alpha subunit p19*), *CLEC5A* (*C‐type lectin domain containing 5A*) and *NPSR1* (*neuropeptide S receptor 1*; Table S2). *STAT* genes are present in several homologues in the genome, and in humans, defects, for example in *STAT3* gene, have been connected with hyper‐IgE syndrome and atopic dermatitis (Boos et al., 2014; Shuai & Liu, 2003) and *NPSR1* has been proposed to be involved in IgE‐mediated diseases in humans, such as allergic eczema and asthma (Acevedo et al., 2017). *CLEC5A* has been connected with innate immunity against several pathogens (Chen et al., 2017) and *IL23A* with inflammation, immune response and cell differentiation and survival (Brocker et al., 2010). However, different studies have detected several and commonly non‐overlapping genomic regions that are associated with IBH (e.g., Shrestha et al., 2015) and various results of the association of IgE levels with IBH. For example, in a study of Kladruber horses (an old Czech horse breed) by Vychodilova et al. (2013), there was no difference between IgE levels in IBH affected versus non‐affected horses, but in Icelandic horses studied by Wilson et al. (2006), a significant difference was found. This might be due to existence of breed‐specific genetic associations (Shrestha et al., 2015). Thus, immune system‐related genes found in our study can be regarded as candidate genes worth of detailed studies in relation to the summer eczema in Finnhorses.

Further, our *F*_ST_ outlier analyses detected SNPs located in genomic regions containing several olfactory receptor‐like genes. Previous studies have found large numbers of non‐synonymous variants in horse olfactory receptor (and immunity‐related) genes, and these loci have turned up in selection studies as well (e.g., Fawcett et al., 2019; Gurgul et al., 2019; Metzger et al., 2014; Orlando et al., 2013). These genes are assumed to be important in mammalian behaviour—for example to find food and mates and to evaluate the environment (Issel‐Tamer & Rine, 1997; Niimura et al., 2014), and they seem to appear consistently as targets of selection in equine studies.

## 5 CONCLUSIONS

We found evidence of selection in the Finnhorse when compared to other breeds in several regions of the genome. Many of the genes at these regions affect height, movement, performance, colour, sugar metabolism, immune response and olfaction and are connected with signal transduction, transcriptional regulation, cell death and cellular responses. On the contrary, selection has not yet resulted in strong differentiation among the breeding sections within the breed. We found strong evidence of divergence in one region, where *DMRT3* gene is located, and only between two breeding sections, the harness trotters and pony‐sized horses. Thus, selection for over 100 years since the breed was founded has induced differentiation through selection. However, time since foundation of the breeding sections has been too short or selection too weak for differences to emerge. This can also be due to acceptance of new horses into the breeding sections from the common gene pool and acceptance of individuals to more than one breeding section. On the other hand, this practice helps to maintain inbreeding at a lower level and effective population sizes higher than they would be without this practice, which are both matters of concern in the breed.

## CONFLICT OF INTEREST

The authors declare no conflict of interest.

## Supporting information

Supplementary Material

## Data Availability

The SNP data are openly available at the European Variation Archive by accession number PRJEB38010 (https://www.ebi.ac.uk/ena/browser/view/PRJEB38010).
