# Supplementary material for: Selection in the Finnhorse, a native all‐around horse breed
Source: J Anim Breed Genet. 2020 Nov 23;138(2):188–203. doi: 10.1111/jbg.12524 (PMC7894145; doi:10.1111/jbg.12524)

Supporting information

Figure S1. Questionnaire for the horse owners


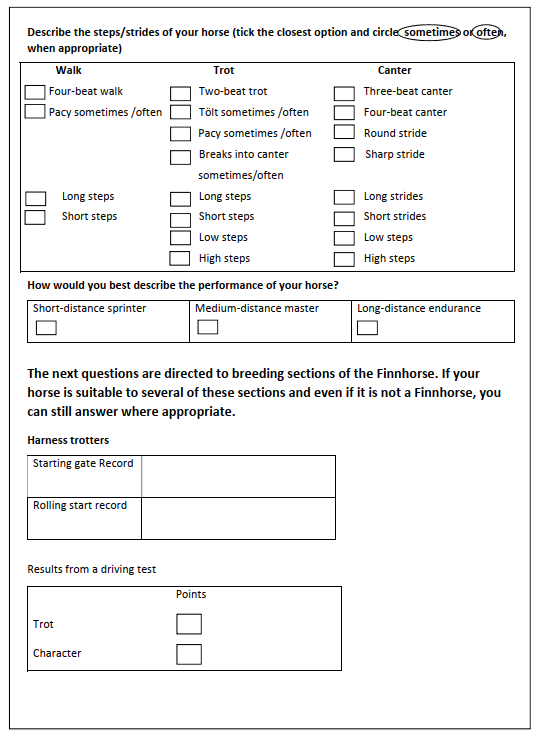


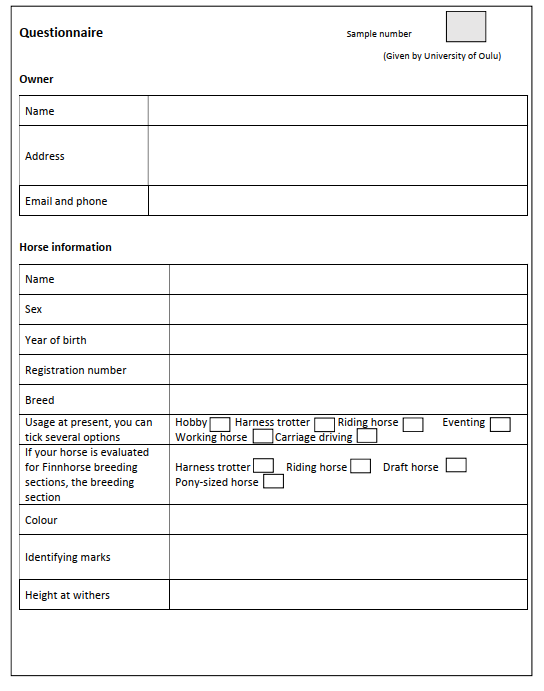

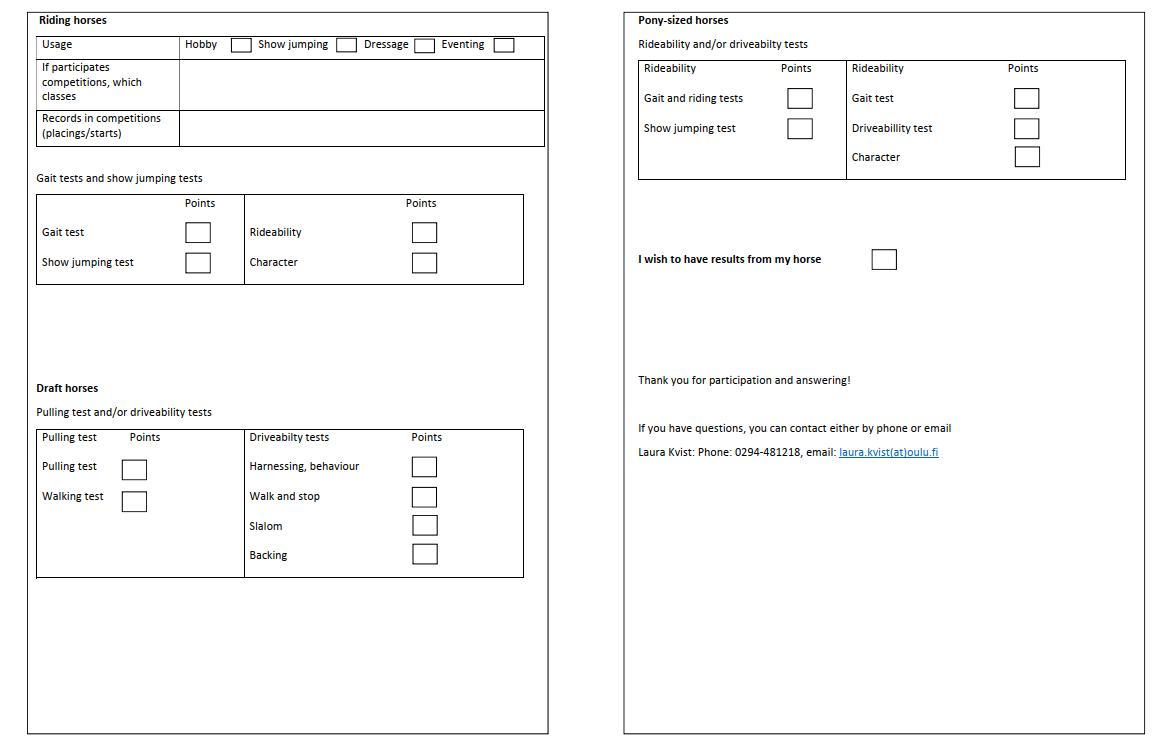


Table S1. PCR and sequencing conditions for selected trait loci.

| Gene | Primers | PCR /sequencing reaction conditions |
| --- | --- | --- |
| *MITF*, *melanogenesis associated transcription factor* | MITF-prom1-forward (labelled with FAM)  5’-CACCGGAAACTTTATCACAGC-3’  MITF-prom1-reverse  5’-AAACTCGTAGGGCTTCCAAAA-3’  (Negro et al. 2017) | 10 μl reactions including 0.5 μl of both primers (10μM), 1 μl of dNTPs (2mM), 2 μl of 5 x HF buffer, 0.1 μl of Phusion polymerase (2U/µl, ThermoFisher) and 10-50 ng of template DNA.  The reaction profile included an initial denaturation at 98 °C for 30 s followed by 35 cycles of 98 °C for 10 s, 59 °C for 30 s and 72 °C for 30 s, finishing with a final extension for 10 min at 72 °C. |
| Microsatellite *TKY284* | TKY284F (labelled with PET)  5’-CTGGACTAGAGTCAGATTGC3’  TKY284R  5’-AACAGGATTCCCCCAATGCC-3’  (Tozaki et al. 2000) | 10 μl reactions including 0.5 μl of both primers (10μM), 1 μl of dNTPs (2mM), 2 μl of 5 x HF buffer, 0.1 μl of Phusion polymerase (2U/µl, ThermoFisher) and 10-50 ng of template DNA.  The reaction profile included an initial denaturation at 98 °C for 30 s followed by 35 cycles of 98 °C for 10 s, 53 °C for 30 s and 72 °C for 30 s, finishing with a final extension for 10 min at 72 °C. |
| *PMEL,* *premelanosome protein* | PMEL-F  5’-ACCAGTTTCCTTCTTCTTCTCCC-3’  PMEL-R  5’-CTCAGACCTGCTGCCCACTGA-3’  Primers designed for this study. | 10 μl reactions including 0.5 μl of both primers (10μM), 1 μl of dNTPs (2mM), 2 μl of 5 x HF buffer, 0.1 μl of Phusion polymerase (2U/µl, ThermoFisher) and 10-50 ng of template DNA.  The reaction profile included an initial denaturation at 98 °C for 30 s followed by 35 cycles of 98 °C for 10 s, 53 °C for 30 s and 72 °C for 30 s, finishing with a final extension for 10 min at 72 °C.  Sequencing was performed with primer *PMEL*-R using BigDye Terminator v.3.1 by diluting the reaction mixture to 1:20. |
| *MSTN*, *myostatin* | MSTN-F  5’-GTAATCAGGTTATAATGCACC-3’  MSTN-R  5’-TTTATTTTGGTTCCCCAAGATTGT-3’  Primers designed for this study. | 10 μl reactions including 0.5 μl of both primers (10μM), 1 μl of dNTPs (2mM), 2 μl of 5 x HF buffer, 0.1 μl of Phusion polymerase (2U/µl, ThermoFisher) and 10-50 ng of template DNA.  The reaction profile included an initial denaturation at 98 °C for 30 s followed by 35 cycles of 98 °C for 10 s, 59 °C for 30 s and 72 °C for 30 s, finishing with a final extension for 10 min at 72 °C.  PCR products were sequenced with the primer MSTN-F using BigDye Terminator v.3.1 by diluting the reaction mixture to 1:50. |
| *DMRT3, double-sex and mab-3-related transcription factor 3* | DMRT3-F  5’-CCTCGGCCGCCGACCGAACT-3’  DMRT3-R  5’-AGAGGAGATGGGGTAGAGCTC-3’  Primers designed for this study. | 10 μl reactions including 0.5 μl of both primers (10μM), 1 μl of dNTPs (2mM), 2 μl of 5 x HF buffer, 0.1 μl of Phusion polymerase (2U/µl, ThermoFisher) and 10-50 ng of template DNA.  The PCR touchdown profile was as follows: 98 °C for 30 s followed by 5 cycles of 98 °C for 10 s, 61 °C for 30 s and 72 °C for 30 s, then 2 cycles of 98 °C for 10 s, 59 °C for 30 s and 72 °C for 30 s and 29 cycles of 98 °C for 10 s, 57 °C for 30 s and 72 °C for 30 s, finishing with 72 °C for 10 min.  PCR products were sequenced with the primer DMRT3-R using BigDye Terminator v.3.1 by diluting the reaction mixture to 1:50 |

References

Negro S., Imsland F., Valera M., Molina A., Solé M. & Andersson L. (2017) Association analysis of KIT, MITF, and PAX3 variants with white markings in Spanish horses. *Animal Genetics* **48**, 349–352.

Tozaki, T., Kakoi, H., Mashima, S., Hirota, K., Hasegawa, T., Ishida, N., Miura, N. & Tomita, M. (2000) The isolation and characterization of 18 equine microsatellite loci, TKY272–TKY289. *Animal Genetics*, **31**, 149.

Table S2. Outlier SNPs from selection tests and genes located close to the SNPs.

1. List of outlier loci between Finnhorses versus other breeds suspect of being under selection by analysis with BayeScan. Altogether 37445 loci that remained after filtering with MAF and linkage disequilibrium were used and 42 outlier loci were found. SNP = name of the SNP in the Illumina chip (bold = same SNP detected also by Arlequin, see below), *F_ST_* = *F_ST_*-value for the SNP, Chr# = number of the chromosome, where the SNP is located, Chr posit. = nucleotide number of the location of the SNP (EquCab2.0, Accession number PRJNA18661), Downstream = genes located close downstream of the SNP, Upstream = genes located close upstream of the SNP, with information of gene functions when available. Information was gathered during 23.3 – 27.3 2020 from GeneCards (<https://www.genecards.org/>), OMIM (<https://www.omim.org/>) and GenBank (<https://www.ncbi.nlm.nih.gov/genbank/>) and from references used in the main text. Genes in bold are of special interest and have been discussed in more detail in the text, some are marked as possible candidates for laminitis and summer eczema, also in bold.

| SNP | *F_ST_* | Cr # | Chr posit. | Downstream | Upstream |
| --- | --- | --- | --- | --- | --- |
| *BIEC2_10058* | 0.1383 | 1 | 22 118 816 | *ADD3*; *Adducin 3*, assembly of spectrin-actin network in erythrocytes and at sites of cell-cell contact in epithelial tissues  *XPNPEP1*; *X-Prolyl Aminopeptidase 1*, catalyzes cleavage of the N-terminal amino acid from a proline residue | *SORCS1*; *Sortilin Related VPS10 Domain Containing Receptor 1*, fasting insulin levels, high diabetes risk, **Laminitis?** |
| ***BIEC2_16328*** | 0.1676 | 1 | 35 865 364 | *CEP55*; *Centrosomal Protein 55*, regulator of the final stages of mitosis  *MYOF*; *myoferlin*, calcium-dependent membrane fusion events | *CYP26A1*; *cytochrome P450 family 26 subfamily A*, member 1 monooxygenases, catalyses reactions involved in drug metabolism and synthesis of [cholesterol](https://en.wikipedia.org/wiki/Cholesterol), [steroids](https://en.wikipedia.org/wiki/Steroid) and other [lipids](https://en.wikipedia.org/wiki/Lipid).  *EXOC6*; *Exocyst complex component 6*, 5' portion of this gene and two neighbouring cytochrome p450 genes are included in a deletion that results in an autosomal dominant form of nonsyndromic optic nerve aplasia. |
| *BIEC2_89368* | 0.1133 | 1 | 182 567 611 | *MDGA2*; *MAM domain containing glycosylphosphatidylinositol anchor 2*, may affect size and muscle phenotype | *RPS29*; *ribosomal protein S29*  *LRR1*; *leucine rich repeat protein 1* |
| ***BIEC2_456242*** | 0.1573 | 2 | 11 504 001 | *LOC100064020*; *cytochrome P450 4A6*  In *cytochrome P450 4B1-like* | *LOC100064092*; *cytochrome P450 4A7-like* |
| ***BIEC2_808557*** | 0.1533 | 3 | 4 840 871 | *SALL1*; *Spalt Like Transcription Factor* 1, diseases connected to this gene include Townes-Brocks Syndrome 1 and Townes-Brocks Syndrome. Connected with pathways for transcriptional regulatory network in embryonic stem cells and developmental biology | *TOX 3*; *TOX high mobility group box family member 3*, connected with breast cancer and restless legs syndrome. Among pathways for ectoderm differentiation and mesodermal commitment. |
| ***BIEC2_795510*** | 0.1353 | 3 | 80 222 892 | ***KIT***; ***proto-oncogene receptor tyrosine kinase***, involved in white coat markings, about 2.4 Mb downstream  *CWH43*; *cell wall biogenesis 43 C-terminal homolog*  *OCIAD1*; *OCIA domain containing 1* | *FRYL*; *FRY like transcription coactivator*, involved in retinitis pigmentosa |
| ***BIEC2_808545*** | 0.1451 | 3 | 105 547 002 | *SLIT2*; *slit guidance ligand 2*, encodes a member of glycoproteins, which are ligands for the Robo family of immunoglobulin receptors. **Summer eczema?** | ***LCORL***; ***ligand dependent nuclear receptor corepressor like***, **connected with body size (height at withers)** |
| *BIEC2_851037* | 0.1281 | 3 | 111 825 382 | In *CLNK*; *cytokine dependent hematopoietic cell linker*, role in immunoreceptor signalling | *WDR1*; *WD repeat domain 1*, neutrophil mediated immunity, e.g. maintenance of epithelial cell polarity |
| *BIEC2_810887* | 0.1275 | 3 | 115 294 618 | In *SORCS2*; *sortilin related VPS10 domain containing receptor 2*, involved in cellular trafficking, associated with deafness and Huntington disease | *AFAP1*; *actin filament associated protein 1* |
| ***BIEC2-860838*** | 0.1524 | 4 | 41 834 018 | *GLCCI1*; *glucocorticoid induced 1*, may be an early marker for glucocorticoid-induced apoptosis, suspect with ichtyosis  In *ICA1*; *islet cell autoantigen 1*, an autoantigen in insulin-dependent diabetes mellitus and primary Sjogren's syndrome. **Laminitis?** | *NXPH1*; *neurexophilin 1*, involved in adhesion between dendrites and axons |
| ***BIEC2_868456*** | 0.1598 | 4 | 63 868 857 | *BBS9*; *Bardet-Biedl syndrome 9*, parathyroid hormone action in bones. Bardet–Biedl syndrome is a human disorder, characterized by obesity, retinitis pigmentosa, polydactyly, hypogonadism, and renal failure,  In *BMPER*; *BMP binding endothelial regulator*, associated with a lethal skeletal disorder | *NPSR1*; *neuropeptide S receptor 1*, involved in IgE-mediated diseases. **Summer eczema?** |
| ***BIEC2_915104*** | 0.1229 | 4 | 73 731 661 | *CAV1*; *caveolin 1 and CAV2*, associated with Berardinelli-Seip syndrome, a disorder characterized by paucity of adipose tissue, extreme insulin resistance, hypertriglyceridemia, hepatic steatosis, and early onset of diabetes. **Laminitis?** | *MET proto-oncogene*, *receptor tyrosine kinase.* MET signaling plays a role in gastrulation, development and migration of muscles and neuronal precursors, angiogenesis and kidney formation, participates in wound healing and organ regeneration and tissue remodeling, differentiation and proliferation of hematopoietic cells, may regulate cortical bone osteogenesis, receptor for Listeria internalin inlB, mediating entry of the pathogen into cells |
| ***BIEC2_919891*** | 0.1338 | 4 | 94 890 989 | *CLEC5A*; *C-type lectin domain containing 5A*, functions in cell adhesion, cell-cell signalling, glycoprotein turnover, inflammation and immune response. **Summer eczema?**  *MGAM*; *maltase-glucoamylase* involved in carbohydrate digestion and absorption, digestion of starch **Laminitis?** | In *MGAM2*; *maltase-glucoamylase 2* (putative), carbohydrate digestion and absorption |
| *BIEC2_954982* | 0.1167 | 5 | 46 546 559 | *MCL1*; *MCL1 apoptosis regulator*, *BCL2 family member*  *ADAMTSL4*; *ADAMTS like 4*, apoptosis | *ECM1*; *extracellular matrix protein 1*, associated with hyalinosis cutis et mucosae  *TARS2*; *threonyl-tRNA synthetase 2*, *mitochondrial* |
| ***BIEC2_979959*** | 0.1244 | 5 | 96 663 962 | *ROR1*; *receptor tyrosine kinase-like orphan receptor 1*, involved in neurite growth | *PGM1*; *phosphoglucomutase 1*, glycogen storage, **Laminitis?** |
| ***BIEC2_1182959*** | 0.1515 | 6 | 70 958 639 | *HOXC4*; *homeobox C4* and several other homeobox genes, morphogenesis | *SMUG1*; *single-strand-selective monofunctional uracil-DNA glycosylase 1*, base excision repair, associated with immunodefiency connected with low or absent serum IgG, IgA, and IgE levels |
| ***BIEC2_1184931***  ***&***  ***BIEC2-1184957*** | 0.1911  0.1939 | 6  6 | 73 997 050  74 046 550 | ***PMEL premelanosome protein*** connected with silver coat colour, ca 300 kb downstream  STAT2; *signal transducer and activator of transcription 2*, connected with **immune system**  *APOF*; *apolipoprotein F*, cholesterol metabolism  In ***IL23A***; ***interleukin 23***, ***alpha subunit p19***, **immunity** | *TIMELESS*, circadian rhythm  *MIP*; *major intrinsic protein of lens fiber*, intracellular communication |
| ***BIEC2_1186739*** | 0.1650 | 6 | 76 331 853 | *LRIG3*; *leucine rich repeats and immunoglobulin like domains 3* | *SLC16A7*; *solute carrier family 16 member 7*, monocarboxylate transporter |
| ***BIEC2_977830*** | 0.1543 | 7 | 7 053 528 | *MTMR2*; *myotubularin related protein 2* | *MAML2*; *mastermind like transcriptional coactivator 2* |
| ***BIEC2_980227*** | 0.1365 | 7 | 12 041 702 | *ANGPTL5*; *angiopoietin like 5*, *CEP126*; *centrosomal protein 126*, microtubules and mitotic spindle organization | *CFAP300*; *cilia and flagella associated protein 300*  *YAP1*; *Yes associated protein 1* |
| ***BIEC2_1003253*** | 0.1323 | 7 | 57 801 333 | *GRM5*; *glutamate metabotropic receptor 5*, regulation of neural network activity and synaptic plasticity | *CTSC*; *cathepsin C*, immune system  *RAB38*; *RAB38*, *member RAS oncogene family*, may be involved in **melanosmal transport** |
| ***BIEC2_1062872*** | 0.1263 | 7 | 79 362 503 | *In SBF2*; *SET binding factor* | *AMPD3*; *adenosine monophosphate deaminase 3*, involved with adenylate catabolic pathway, erythrocyte isoforms |
| ***BIEC2_1018152*** | 0.1298 | 7 | 94 907 521 | *KIF18A*; *kinesin family member 18A*, actin binding and microtubule motor activity  *METTL15*; *methyltransferase like 15* |  |
| ***BIEC2_1055827*** | 0.1168 | 8 | 59 913 391 | No known genes close |  |
| ***TBIEC2_1137029*** | 0.1620 | 9 | 18 056 124 | *CPA6*; *carboxypeptidase A6*, a preprotein for a mature enzyme that functions from digestion of food to selective biosynthesis of neuroendocrine peptides  In *ARFGEF1*; *ADP ribosylation factor guanine nucleotide exchange factor 1*, involved in intracellular vesicular trafficking | *CSPP1*; *centrosome and spindle pole associated protein 1*, role in cell-cycle progression and spindle organization |
| *BIEC2_1091610* | 0.1224 | 9 | 45 861 396 | *VPS13B*; *vacuolar protein sorting 13 homolog B*, involved in development and the function of the eye, hematological system and central nervous system  In *RGS22*; *regulator of G protein signaling 22*, inhibits signal transduction | *FBXO43*; *F-box protein 43*, involved in meiosis |
| ***BIEC2_124553*** | 0.1656 | 10 | 53 830 445 | ***MCHR2 melanin concentrating hormone receptor 2***, **control of feeding behaviors and energy metabolism. Coat colour?**  *SIM1*; *single-minded family bHLH transcription factor 1*  *ASCC3*; *activating signal cointegrator 1 complex subunit 3*, ATP-dependent unwinding of nucleic acid duplexes  *GRIK2*; *Glutamate ionotropic receptor kainate type subunit 2*, neurotransmission in the brain, associated with learning and memory | *HACE1*; *HECT domain and ankyrin repeat containing E3 ubiquitin protein ligase 1*, tagging and subcellular localization or proteasomal degradation of proteins |
| ***BIEC2_190039*** | 0.1151 | 12 | 19 208 642 | *FAM111A*; *family with sequence similarity 111 member A*, **involved in bone development**  In *DTX4*; *deltex E3 ubiquitin ligase 4*, cell-cell communication | *MPEG1*; *macrophage expressed 1*, immune response *LOC100068504*; *olfactory receptor 5A1-like*, *LOC100068543*; *olfactory receptor 1440-like*, *LOC100068520*; *olfactory receptor 1440-like* |
| ***BIEC2_235072*** | 0.1344 | 13 | 36 899 557 | In *RBFOX1*; *RNA binding fox-1 homolog 1*, neurodevelopment, skeletal muscle mass |  |
| ***BIEC2_242918*** | 0.1522 | 14 | 9 316 928 | *STK10*; *serine/threonine kinase 10*, *immune system* In ***FBXW11***; *F-box and WD repeat domain containing 11*, associated with **immune system and height** | ***SMIM23***, ***small integral membrane protein 23***, **migration of neural crest cells**, **associated with hearing loss and pigmentation changes in humans (like *PAX3* and *MITF*)** |
| ***BIEC2_271112*** | 0.1500 | 14 | 83 458 785 | *XRCC4 X-ray repair cross complementing 4*, repair of DNA double-strand breaks  *TMEM167A*; *transmembrane protein 167A* |  |
| *BIEC2-335427* | 0.1144 | 15 | 75 586 809 | *tRNA-Ala* | *OSR1*; *odd-skipped related transcription factor 1*, regulation of embryonic heart and urogenital development |
| ***BIEC2-332488*** | 0.1507 | 16 | 21 084 820 | ***MITF***; ***Microphthalmia-associated transcription factor or melanogenesis associated transcription factor***, **white coat colour variants and markings in horses ‘splashed white’**  *FRMD4B*; *FERM domain containing 4B*, protein scaffolding  In *FAM19A4*; *family with sequence similarity 19 (chemokine (C-C motif)-like)*, *member A4* | *FAM19A1*; *family with sequence similarity 19 (chemokine (C-C motif)-like)*, *member A1* |
| ***BIEC2_337274*** | 0.1330 | 16 | 28 745 680 | In *FHIT*; *fragile histidine triad*, *purine metabolism* |  |
| ***BIEC2_439076*** | 0.1231 | 18 | 67 377 787 | ***MSTN***; ***myostatin***, **performance** < 1 Mb downstream  *HIBCH*; *3-hydroxyisobutyryl-CoA hydrolase*  *INPP1*; *inositol polyphosphate-1-phosphatase*, *phosphatidylinositol signaling*  *MFSD6*; *major facilitator superfamily domain containing 6*  *NEMP2*; *nuclear envelope integral membrane protein 2*  *NAB1*; *NGFI-A binding protein 1*  *GLS*; *glutaminase*, hydrolysis of glutamine to glutamate and ammonia  ***STAT1***; *signal transducer and activator of transcription 1*  In ***STAT4***; *signal transducer and activator of transcription 4*, both associated with immune system. **Summer eczema?** | *MYO1B*; *myosin IB*, neuronal development and function |
| ***BIEC2_430816*** | 0.1168 | 19 | 18 728 881 | *NDUFB5*; *NADH:ubiquinone oxidoreductase subunit B5*, electron transport to respiratory chain  In *USP13*; *ubiquitin specific peptidase 13 (isopeptidase T-3)* | *PEX5L*; *peroxisomal biogenesis factor 5 like*, role in cyclic nucleotide-gated (HCN) channels |
| ***BIEC2_566326*** | 0.1524 | 21 | 42 003 047 | *CDH18*; *cadherin 18*, cell-cell adhesion |  |
| ***BIEC2_575339*** | 0.1236 | 22 | 1 496 512 | *SYNDIG1*; *synapse differentiation inducing 1* |  |
| ***BIEC2_606057*** | 0.1317 | 23 | 3 579 451 | *DAPK1*; *death associated protein kinase 1*, gamma-interferon induced programmed cell death | *LOC106782456*; *formin-like protein 18*, morphogenesis, cytokinesis, and cell polarity  *TUT7*; *terminal uridylyl transferase 7*, oocyte maturation and fertility |
| ***BIEC2_858275*** | 0.1178 | 30 | 7 086 755 | *ITPK*;*B inositol-trisphosphate 3-kinase B*, synthesis of inositol tetraphosphate  *STUM*; *stum*, *mechanosensory transduction mediator homolog* | *PARP1*; *poly(ADP-ribose) polymerase 1*, modifies nuclear proteins by poly(ADP-ribosyl)ation. |
| ***BIEC2_872929*** | 0.1663 | 31 | 171 187 | *TMEM242*; *transmembrane protein 242*  In *ZDHHC14*; *zinc finger DHHC-type palmitoyltransferase 14* | *SNX9*; *sorting nexin 9*, intracellular trafficking |

b) List of outlier loci detected by BayeScan between breeding sections of the Finnhorse.

| SNP | F_ST_ | | Chr # | | Chr posit. | | Downstream | | Upstream | |
| --- | --- | --- | --- | --- | --- | --- | --- | --- | --- | --- |
| Trotters vs. pony-sized horses | | | | | | |  | |  | |
| *BIEC2_620406* | 0.1225 | | 23 | | 23 462 382 | | ***DMRT3***; *Doublesex And Mab-3 Related Transcription Factor 3*, **configures spinal circuits controlling stride in vertebrates** | | *SMARCA2*; *SWI/SNF related, matrix associated, actin dependent regulator of chromatin, subfamily A, member 2*, regulates transcription by altering the chromatin structure | |
| Trotters vs. draught horses | | | | | | | |  | |  |
| None | |  | |  | |  | |  | |  |
|  | |  | |  | |  | |  | |  |
| Trotters vs. riding horses | | | | | | | |  | |  |
| None | |  | |  | |  | |  | |  |
| Riding vs. draught horses | | | | | | | |  | |  |
| None | | | | | | | | | | |
|  | |  | |  | |  | |  | |  |
| Riding vs. pony-sized horses | | | | | | | |  | |  |
| None | |  | |  | |  | |  | |  |
|  | |  | |  | |  | |  | |  |
| Draught vs. pony-sized horses | | | | | | | |  | |  |
| None | |  | |  | |  | |  | |  |

1. List of the 55 outlier loci between Finnhorses versus other breeds suspect of being under selection by analysis with Arlequin. Loci with F_ST_ > 0.5 and a significant p-value are listed. NA = p-value not computed value due to Obs. Het. BP was out of simulated bounds. Bolded SNP names were detected also by BayeScan.

| SNP | F_ST_  (p-value) | Chr # | Chr posit. | Downstream | Upstream |
| --- | --- | --- | --- | --- | --- |
| ***BIEC2_16328*** | 0.5712  (0.00067) | 1 | 35 835 357 | *CEP55*; *Centrosomal Protein 55*, regulator of the final stages of mitosis  *MYOF*; *myoferlin*, calcium-dependent membrane fusion events | *CYP26A1*; *cytochrome P450 family 26 subfamily A member 1 monooxygenases*, catalyses reactions involved in drug metabolism and synthesis of cholesterol, steroids and other lipids  *EXOC6*; *Exocyst complex component 6*, 5' portion of this gene and two neighbouring cytochrome p450 genes are included in a deletion that results in an autosomal dominant form of nonsyndromic optic nerve aplasia. |
| *BIEC2_61094* | 0.5160  (0.00275) | 1 | 138 929 107 | *In DMXL2*; *Dmx like 2*, Notch signalling pathway, connected e.g. with metabolism, immune system and growth |  |
| *BIEC2_71470* | 0.5308  (0.00310) | 1 | 156 105 494 | *LOC100072271*; *olfactory receptor 11G2-like LOC100072265*; *olfactory receptor 11H6-like* | *LOC106782091*; *olfactory receptor 11H7-like*  *LOC100072282*; *olfactory receptor 11G2* |
| *BIEC2_85106* | 0.5063  (0.00001) | 1 | 176 577 996 | *FBXO33*; *F-box protein 33*, associated with susceptibility to osteoporosis and ADHD | *LRFN5*; *leucine rich repeat and fibronectin type III domain containing 5* |
| ***BIEC2_456242*** | 0.6642  (0.00000) | 2 | 11 504 001 | *LOC100064020*; *cytochrome P450 4A6*  In *cytochrome P450 4B1-like* | *LOC100064092*; *cytochrome P450 4A7-like* |
| *BIEC2_500003* | 0.5021  (0.00293) | 2 | 95 032 492 | *PCDH18*; *protocadherin 18*, associated with e.g. cardiovascular system and size | *PABPC4L*; *poly(A) binding protein cytoplasmic 4 like* |
| ***BIEC2_808557*** | 0.5214  (0.00103) | 3 | 4 840 871 | *SALL1; Spalt Like Transcription Factor 1*, diseases connected to this gene include Townes-Brocks Syndrome 1 and Townes-Brocks Syndrome. Connected with pathways for transcriptional regulatory network in embryonic stem cells and developmental biology | *TOX 3*; *TOX high mobility group box family member 3*, this gene has been connected with breast cancer and restless legs syndrome. Among pathways for ectoderm differentiation and mesodermal commitment |
| ***BIEC2_795510*** | 0.5659  (0.00054) | 3 | 80 222 892 | ***KIT***; ***proto-oncogene receptor tyrosine kinase***, **involved in white coat markings**, about 2.4 Mb downstream  *CWH43*; *cell wall biogenesis 43 C-terminal homolog*  *OCIAD1*; *OCIA domain containing 1* | *FRYL*; *FRY like transcription coactivator*, involved in retinitis pigmentosa |
| ***BIEC2_808545*** | 0.6084  (0.14551) | 3 | 105 547 002 | *SLIT2*; *slit guidance ligand 2*, encodes a member of glycoproteins, which are ligands for the Robo family of immunoglobulin receptors. **Summer eczema?** | ***LCORL***; ***ligand dependent nuclear receptor corepressor like***, **connected with body size (height at withers)** |
| *BIEC2_852529* | 0.5098  (0.00140) | 4 | 20 572 040 | *DDC*; *dopa decarboxylase*, involved in dopamine metabolism  In *GRB10*; *growth factor receptor bound protein 10*, associated with growth, included in KIT receptor signalling pathway | *COBL*; *cordon-bleu WH2 repeat protein*, role in actin cytoskeleton |
| *BIEC2_896783* | 0.5000  (0.00294) | 4 | 20 952 802 | *GRB10*; *growth factor receptor bound protein 10*, associated with growth, included in KIT receptor signalling pathway  In *COBL*; *cordon-bleu WH2 repeat protein*, role in actin cytoskeleton |  |
|  |  |  |  |  |  |
| ***BIEC2-860838*** | 0.5214  (0.00102) | 4 | 41 834 018 | *GLCCI1*; *glucocorticoid induced 1*, may be an early marker for glucocorticoid-induced apoptosis, suspect with ichtyosis.  In *ICA1*; *islet cell autoantigen 1*, the protein is believed to be an autoantigen in insulin-dependent diabetes mellitus and primary Sjogren's syndrome. **Laminitis?** | *NXPH1*; *neurexophilin 1*, involved in adhesion between dendrites and axons |
| *BIEC2_862159* | 0.5080  (0.00078) | 4 | 44 032 421 |  | *NDUFA4*; *NDUFA4 mitochondrial complex associated*, involved in mitochondrial respiratory chain  *PHF14*; *PHD finger protein 14* |
| ***BIEC2_868456*** | 0.6542  (0.00000) | 4 | 63 868 857 | *BBS9*; *Bardet-Biedl syndrome 9*, parathyroid hormone action in bones. Bardet–Biedl syndrome is a human disorder, characterized by obesity, retinitis pigmentosa, polydactyly, hypogonadism, and renal failure  In *BMPER*; *BMP binding endothelial regulator*, associated with a lethal skeletal disorder | *NPSR1*; *neuropeptide S receptor 1*, *involved in IgE-mediated diseases*. ***Summer eczema?*** |
| ***BIEC2_915104*** | 0.5414  (0.00163) | 4 | 73 731 661 | *CAV1*; *caveolin 1 and CAV2*, associated with Berardinelli-Seip syndrome, a disorder characterized by paucity of adipose tissue, extreme insulin resistance, hypertriglyceridemia, hepatic steatosis, and early onset of diabetes. **Laminitis?** | *MET proto-oncogene*, *a receptor tyrosine kinase*. MET signaling plays a role in gastrulation, development and migration of muscles and neuronal precursors, angiogenesis and kidney formation, participates in wound healing and organ regeneration and tissue remodeling, differentiation and proliferation of hematopoietic cells, may regulate cortical bone osteogenesis, receptor for Listeria internalin inlB, mediating entry of the pathogen into cells |
| ***BIEC2_919891*** | 0.5691  (0.00249) | 4 | 94 890 989 | *CLEC5A*; *C-type lectin domain containing 5A*, functions in cell adhesion, cell-cell signalling, glycoprotein turnover, inflammation and immune response. **Summer eczema?**  *MGAM*; *maltase-glucoamylase*, involved in carbohydrate digestion and absorption, digestion of starch **Laminitis?** | *In MGAM2*; *maltase-glucoamylase 2* (putative), carbohydrate digestion and absorption |
| *BIEC2_881825* | 0.5160  (0.00275) | 4 | 103 436 292 | *PRKAG2*; *protein kinase AMP-activated non-catalytic subunit gamma 2*, fatty acid and cholesterol biosynthesis  In *GALNTL5*; *polypeptide N-acetylgalactosaminyltransferase like 5* | *GALNT11*; *polypeptide N-acetylgalactosaminyltransferase 11*, role e.g. in carbohydrate binding |
| *BIEC2_908009* | 0.5066  (0.00028) | 5 | 47 955 881 | *PEX11B*; *peroxisomal biogenesis factor 11 beta*, peroxisomal proliferation  In *ITGA10*; *integrin subunit alpha 10*, cell adhesion and cell-surface mediated signalling | *ANKRD35*; *ankyrin repeat domain 35* |
| ***BIEC2_979959*** | 0.5501  (0.00056) | 5 | 96 663 962 | *ROR1*; *receptor tyrosine kinase-like orphan receptor 1*, involved in neurite growth | *PGM1*; *phosphoglucomutase 1*, glycogen storage, Laminitis? |
| ***BIEC2_1182959*** | 0.5204  (0.00102) | 6 | 70 958 639 | *HOXC4*; *homeobox C4* and several other homeobox genes | *SMUG1*; *single-strand-selective monofunctional uracil-DNA glycosylase 1* |
| ***BIEC2_1184931***  ***&***  ***BIEC2-1184957*** | 0.6577  (0.00040)  0.6577  (0.00040) | 6  6 | 73 997 050  74 046 550 | *PMEL premelanosome protein* connected with silver coat colour ca 300 kb downstream  *STAT2*; *signal transducer and activator of transcription 2*, connected with immune system  *APOF*; *apolipoprotein F*, cholesterol metabolism  In *IL23A*; *interleukin 23*, *alpha subunit p19*, immunity | *TIMELESS*, circadian rhythm  *MIP*; *major intrinsic protein of lens fiber*, intracellular communication |
| ***BIEC2_1186739*** | 0.5712  (0.00068) | 6 | 76 331 853 | *LRIG3*; *leucine rich repeats and immunoglobulin like domains 3* | *SLC16A7*; *solute carrier family 16 member 7*, monocarboxylate transporter |
| ***BIEC2_977830*** | 0.5214  (0.00103) | 7 | 7 053 528 | *MTMR2 myotubularin related protein 2* | *MAML2*; *mastermind like transcriptional coactivator 2* |
| ***BIEC2_980227*** | 0.56610  (0.00055) | 7 | 12 041 702 | *ANGPTL5*; *angiopoietin like 5*  *CEP126*; *centrosomal protein 126*, microtubules and mitotic spindle organization | *CFAP300*; *cilia and flagella associated protein 300*, *YAP1*; *Yes associated protein 1* |
| *BIEC2_995757* | 0.5246  (0.00052) | 7 | 36 852 063 |  | *ETS1*; *ETS proto-oncogene 1*, transcription factor involved in stem cell development, cell senescence and death, and tumorigenesis. |
| ***BIEC2_1003253*** | 0.5713  (0.00019) | 7 | 57 801 333 | *GRM5*; *glutamate metabotropic receptor 5*, regulation of neural network activity and synaptic plasticity | *CTSC*; *cathepsin C*, immune system  *RAB38*; *RAB38*, *member RAS oncogene family*, may be involved in **melanosmal transport** |
| ***BIEC2_1062872*** | 0.5364  (0.00000) | 7 | 79 362 503 | In *SBF2*; *SET binding factor* | *AMPD3*; *adenosine monophosphate deaminase 3*, involved with adenylate catabolic pathway, erythrocyte isoforms |
| ***BIEC2_1018152*** | 0.5226  (0.00000) | 7 | 94 907 521 | *KIF18A*; *kinesin family member 18A*, actin binding and microtubule motor activity  *METTL15*; *methyltransferase like 15* |  |
| ***BIEC2_1055827*** | 0.5289  (0.00051) | 8 | 59 913 391 | No known genes close |  |
| *UKUL1925* | 0.5160  (0.00275) | 9 | 6 385 086 | *FABP12*; *fatty acid binding protein 12*, lipoprotein and fatty acid uptake, transport and metabolism, **obesity**, **laminitis?** | *FABP4*; *fatty acid binding protein 4*, lipoprotein and fatty acid uptake, transport and metabolism  **obesity**, **laminitis?** |
| ***TBIEC2_1137029*** | 0.6802  (0.00000) | 9 | 18 056 124 | *CPA6*; *carboxypeptidase A6*, *a* preprotein for a mature enzyme that functions from digestion of food to selective biosynthesis of neuroendocrine peptides  In *ARFGEF1*; *ADP ribosylation factor guanine nucleotide exchange factor 1*, involved in intracellular vesicular trafficking | *CSPP1*; *centrosome and spindle pole associated protein 1*, role in cell-cycle progression and spindle organization |
| ***BIEC2_124553*** | 0.5712  (0.00068) | 10 | 53 830 445 | ***MCHR2 melanin concentrating hormone receptor 2***, **control of feeding behaviors and energy metabolism**. **Coat colour?**  *SIM1*; *single-minded family bHLH transcription factor 1*  *ASCC3*; *activating signal cointegrator 1 complex subunit 3*, ATP-dependent unwinding of nucleic acid duplexes  *GRIK2*; *Glutamate ionotropic receptor kainate type subunit 2*, neurotransmission in the brain, associated with learning and memory | *HACE1*; *HECT domain and ankyrin repeat containing E3 ubiquitin protein ligase 1*, tagging and subcellular localization or proteasomal degradation of proteins |
| ***BIEC2_190039*** | 0.5284  (0.00049) | 12 | 19 208 642 | *FAM111A*; *family with sequence similarity 111 member A*, involved in bone development  In *DTX4*; *deltex E3 ubiquitin ligase 4*, cell-cell communication | *MPEG1*; *macrophage expressed 1*, *immune response*, *LOC100068504*; *olfactory receptor 5A1-like LOC100068543*; *olfactory receptor 1440-like LOC100068520*; *olfactory receptor 1440-like* |
| ***BIEC2_235072*** | 0.5790  (0.00001) | 13 | 36 899 557 | In *RBFOX1*; *RNA binding fox-1 homolog 1*, neurodevelopment, skeletal muscle mass |  |
| ***BIEC2_242918*** | 0.5214  (0.00103) | 14 | 9 316 928 | *STK10*; *serine/threonine kinase 10*, *immune system*  In *FBXW11*; *F-box and WD repeat domain containing 11*, associated with immune system and height | ***SMIM23***, ***small integral membrane protein 23***, **migration of neural crest cells**, **associated with hearing loss and pigmentation changes in humans (like PAX3 and MITF)** |
| *BIEC2_258052* | 0.5242  (0.00195) | 14 | 45 786 698 | *ISOC1*; *isochorismatase domain containing 1* | *SLC27A6*; *solute carrier family 27 (fatty acid transporter)*, *member 6* |
| ***BIEC2_271112*** | 0.5214  (0.00103) | 14 | 83 458 785 | *XRCC4*; *X-ray repair cross complementing 4*, repair of DNA double-strand breaks,  *TMEM167A*; *transmembrane protein 167A* |  |
| *BIEC2_310588* | 0.5158  (0.00278) | 15 | 51 973 518 | *STPG4*; *sperm-tail PG-rich repeat containing 4*, role in epigenetic chromatin reprogramming during early development,  In *TTC7A*; *tetratricopeptide repeat domain 7A*, intestinal development | *MCFD2*; *multiple coagulation factor deficiency 2*, blood coagulation, maintenance of stem cell potential in nervous system |
| *BIEC2_333776* | 0.5158  (0.00278) | 15 | 72 389 304 | *KLHL29*; *kelch like family member 29* |  |
| ***BIEC2-332488*** | 0.5367  (NA) | 16 | 21 084 820 | ***MITF***; ***Microphthalmia-associated transcription factor or melanogenesis associated transcription factor***, **white coat colour variants and markings in horses ‘splashed white’**  *FRMD4B*; *FERM domain containing 4B*, *protein scaffolding*  In *FAM19A4*; *family with sequence similarity 19 (chemokine (C-C motif)-like)*, *member A4* | *FAM19A1*; *family with sequence similarity 19 (chemokine (C-C motif)-like)*, *member A1* |
| ***BIEC2_337274*** | 0.5739 | 16 | 28 745 680 | In *FHIT*; *fragile histidine triad*, purine metabolism |  |
| *BIEC2_394525* | 0.5667  (0.00042) | 17 | 36 139 713 | *TDRD3*; *tudor domain containing 3* | *PCDH20*; *protocadherin 20* |
| ***BIEC2_439076*** | 0.5321  (0.00001) | 18 | 67 377 787 | ***MSTN***; ***myostatin***, **performance** < 1 Mb downstream  *HIBCH*; *3-hydroxyisobutyryl-CoA hydrolase*  *INPP1*; *inositol polyphosphate-1-phosphatase*, *phosphatidylinositol signaling*  *MFSD6*; *major facilitator superfamily domain containing 6*  *NEMP2*; *nuclear envelope integral membrane protein 2*  *NAB1*; *NGFI-A binding protein 1*  *GLS*; *glutaminase*, hydrolysis of glutamine to glutamate and ammonia  ***STAT1***; *signal transducer and activator of transcription 1*  In ***STAT4***; *signal transducer and activator of transcription 4*, both associated with immune system. **Summer eczema?** | *MYO1B*; *myosin IB*, neuronal development and function |
| ***BIEC2_430816*** | 0.5163  (0.00152) | 19 | 18 728 881 | *NDUFB5*; *NADH:ubiquinone oxidoreductase subunit B5*, electron transport to respiratory chain  In *USP13*; *ubiquitin specific peptidase 13 (isopeptidase T-3)* | *PEX5L*; *peroxisomal biogenesis factor 5 like*, role in cyclic nucleotide-gated (HCN) channels |
| *BIEC2_444949* | 0.5192  (0.00224) | 19 | 51 921 242 | *ALCAM*; *activated leukocyte cell adhesion molecule*, immunoglobulin receptor |  |
| ***BIEC2_566326*** | 0.5214  (0.00103) | 21 | 42 003 047 | *CDH18*; *cadherin 18*, cell-cell adhesion |  |
| ***BIEC2_575339*** | 0.5722  (0.00230) | 22 | 1 496 512 | *SYNDIG1*; *synapse differentiation inducing 1* |  |
| *BIEC2_590198* | 0.5115  (0.00324) | 22 | 27 376 449 | ***SOGA1***; *suppressor of glucose*, *autophagy associated 1*, regulates autophagy by reduction of glucose production in an adiponectin- and insulin-dependent manner. **Laminitis?**  *TLDC2*; *TBC/LysM-associated domain containing 2*  In ***SAMHD1***; *SAM and HD domain containing deoxynucleoside triphosphate triphosphohydrolase 1*, role in innate **immune response** | *RBL1*; *RB transcriptional corepressor like 1*, cell cycle regulation |
| *BIEC2_590990* | 0.5251  (0.00193) | 22 | 29 326 274 | *DHX35*; *DEAH-box helicase 35* |  |
| ***BIEC2_606057*** | 0.5685  (0.00252) | 23 | 3 579 451 | *DAPK1*; *death associated protein kinase 1*, gamma-interferon induced programmed cell death | *LOC106782456*; *formin-like protein 18*, morphogenesis, cytokinesis, and cell polarity  *TUT7*; *terminal uridylyl transferase 7*, *oocyte* maturation and fertility |
| *BIEC2_606735* | 0.5194  (0.00224) | 23 | 4 323 051 | *GOLM1*; *golgi membrane protein 1*, protein export from the endoplasmic reticulum  In *NAA35*; *N-alpha-acetyltransferase 35*, *NatC auxiliary subunit*, regulation of apoptosis and proliferation of smooth muscle cells | *AGTPBP1*; *ATP/GTP binding protein 1*, deglutamylation of proteins |
| *UKUL3868* | 0.5276  (0.00306) | 25 | 18 869 454 | In *ZNF618*; *zinc finger protein 618*, transcriptional regulation | *AMBP*; *alpha-1-microglobulin/bikunin precursor*, may play a role in the regulation of inflammatory processes and many physiological and pathological processes.  *KIF12*; *kinesin family member 12*, intracellular transport and cell division |
| ***BIEC2_858275*** | 0.5393  (0.00163) | 30 | 7 086 755 | *ITPK*; *B inositol-trisphosphate 3-kinase B*, synthesis of inositol tetraphosphate  *STUM*; *stum*, mechanosensory transduction mediator homolog | *PARP1*; *poly(ADP-ribose) polymerase 1*, *modifies nuclear proteins by poly(ADP-ribosyl)ation*. |
| ***BIEC2_872929*** | 0.5712  (0.00068) | 31 | 171 187 | *TMEM242*; *transmembrane protein 242*  *In ZDHHC14*; *zinc finger DHHC-type palmitoyltransferase 14* | *SNX9*; *sorting nexin 9*, intracellular trafficking |

d) List of outlier loci detected by Arlequin between breeding sections of the Finnhorse. Loci with FST > 0.6 and a significant p-value are listed. NA = p-value not computed value due to Obs. Het. BP was out of simulated bounds. Bolded SNP name was detected also by BayeScan.

| SNP | F_ST_ | Chr # | Chr posit. | Downstream | Upstream |
| --- | --- | --- | --- | --- | --- |
| Trotters vs. pony-sized horses | | | |  |  |
| *BIEC2_844838* | 0.60791  0.00000 | 4 | 4540307 | *RELN*; *reelin, cell-cell interactions*, neuronal migration | *ORC5*; *origin recognition complex subunit 5*, DNA replication, involved in hyperinsulinemic hypoglycemia in humansd |
| ***BIEC2_620406*** | 0.66173  NA | 23 | 23 462 382 | ***DMRT3***; *Doublesex And Mab-3 Related Transcription Factor 3*, **configures spinal circuits controlling stride in vertebrates** | *SMARCA2*; *SWI/SNF related, matrix associated, actin dependent regulator of chromatin, subfamily A, member 2*, regulates transcription by altering the chromatin structure |
| *BIEC2_639984* | 0.60139  0.00003 | 24 | 24757386 | In *NRXN3*; *neurexin 3*, member of a protein family that functions in the nervous system as receptors and cell adhesion molecules | *DIO2*; *iodothyronine deiodinase 2*, catalyzes the conversion of thyroxine (thyroid hormone |
| *BIEC2_1121455* | 0.60000  0.00000 | X | 38816631 | *LOC102149373*; CX*XC-type zinc finger protein 1-like*, transcription factor | *ZNF81*; *zinc finger protein 81*, transcription factor |
| *TBIEC2_1191635* | 0.602960.00001 | X | 70088207 | *CPXCR1*; *CPX chromosome region, candidate* *1*, connected with cleft palate syndrome | *TGIF2LX*; TG*FB induced factor homeobox 2 like X-linked*, spermatogenesis? |
| Trotters vs. draught horses | | | |  |  |
| None |  |  |  |  |  |
|  |  |  |  |  |  |
| Trotters vs. riding horses | | | |  |  |
| None |  |  |  |  |  |
| Riding vs. draught horses | | | |  |  |
| Observed F-statistics lead to invalid migration rates and coalescent simulations could not be performed | | | | | |
|  |  |  |  |  |  |
| Riding vs. pony-sized horses | | | |  |  |
| None |  |  |  |  |  |
|  |  |  |  |  |  |
| Draught vs. pony-sized horses | | | |  |  |
| None |  |  |  |  |  |

Table S3. Results from gene ontology and enrichment analysis from program DAVID for genes located close to SNPs detected by the selection tests. Altogether 177 genes were found to be enriched, from 1.09 to 135.09 fold, with p-values < 0.05. However, Bonferroni-corrected p-values were not significant. The ontology terms are listed by increasing p-values. The Gene ontology (GO) terms enriched by > 20 fold are marked in bold. These genes represent ten different annotation categories that can be clustered into 1) biological processes (GOTERM_BP_ALL), 2) cellular components (GOTERM_CC_ALL, GOTERM_CC_DIRECT, INTERPRO), 3) molecular functions (GOTERM_MF_ALL, GOTERM_MF_DIRECT), 4) pathways (KEGG_PATHWAY) and 4) EMBL and UNiProt domains (SMART, UP_KEYWORDS). Twenty-four genes of interest were not mapped by DAVID and thus not included in the analysis (for example PMEL).

| Category | Term | P-value | Genes | Fold Enrichment | Bonferroni p-value |
| --- | --- | --- | --- | --- | --- |
| GOTERM_BP_ALL | **GO:0030857~negative regulation of epithelial cell differentiation** | 0.0006 | *CAV1, OSR1, YAP1, STAT1* | 23.4939 | 0.7406 |
| INTERPRO | **IPR012345:STAT transcription factor, DNA-binding, subdomain** | 0.0008 | *STAT4, STAT1, STAT2* | 65.6903 | 0.2427 |
| INTERPRO | **IPR013800:STAT transcription factor, all-alpha** | 0.0008 | *STAT4, STAT1, STAT2* | 65.6903 | 0.2427 |
| INTERPRO | **IPR001217:STAT transcription factor, core** | 0.0008 | *STAT4, STAT1, STAT2* | 65.6903 | 0.2427 |
| INTERPRO | **IPR015988:STAT transcription factor, coiled coil** | 0.0008 | *STAT4, STAT1, STAT2* | 65.6903 | 0.2427 |
| INTERPRO | **IPR013801:STAT transcription factor, DNA-binding** | 0.0008 | *STAT4, STAT1, STAT2* | 65.6903 | 0.2427 |
| INTERPRO | **IPR013799:STAT transcription factor, protein interaction** | 0.0008 | *STAT4, STAT1, STAT2* | 65.6903 | 0.2427 |
| SMART | **SM00964:SM00964** | 0.0010 | *STAT4, STAT1, STAT2* | 57.9773 | 0.0886 |
| UP_KEYWORDS | SH2 domain | 0.0012 | *GRB10, STAT4, STAT1, STAT2* | 18.9879 | 0.0937 |
| GOTERM_BP_ALL | GO:0071375~cellular response to peptide hormone stimulus | 0.0019 | *GRB10, CAV1, SOGA1, MSTN, STAT1, PARP1* | 6.6987 | 0.9862 |
| GOTERM_BP_ALL | GO:1901653~cellular response to peptide | 0.0023 | *GRB10, CAV1, SOGA1, MSTN, STAT1, PARP1* | 6.4329 | 0.9940 |
| GOTERM_BP_ALL | **GO:0072182~regulation of nephron tubule epithelial cell differentiation** | 0.0023 | *OSR1, YAP1, STAT1* | 40.5270 | 0.9941 |
| GOTERM_BP_ALL | **GO:0072160~nephron tubule epithelial cell differentiation** | 0.0023 | *OSR1, YAP1, STAT1* | 40.5270 | 0.9941 |
| GOTERM_BP_ALL | **GO:2000696~regulation of epithelial cell differentiation involved in kidney development** | 0.0033 | *OSR1, YAP1, STAT1* | 33.7725 | 0.9994 |
| GOTERM_BP_ALL | GO:0043434~response to peptide hormone | 0.0038 | *GRB10, CAV1, SOGA1, MSTN, STAT1, PARP1* | 5.7080 | 0.9998 |
| GOTERM_BP_ALL | **GO:0042522~regulation of tyrosine phosphorylation of Stat5 protein** | 0.0046 | *CAV1, IL23A, KIT* | 28.9478 | 1.0000 |
| GOTERM_BP_ALL | GO:0032869~cellular response to insulin stimulus | 0.0055 | *GRB10, SOGA1, MSTN, STAT1, PARP1* | 6.9634 | 1.0000 |
| INTERPRO | IPR000980:SH2 domain | 0.0062 | *GRB10, STAT4, CLNK, STAT1, STAT2* | 6.7722 | 0.8760 |
| GOTERM_BP_ALL | **GO:0042506~tyrosine phosphorylation of Stat5 protein** | 0.0067 | *CAV1, IL23A, KIT* | 23.8394 | 1.0000 |
| GOTERM_BP_ALL | GO:1901652~response to peptide | 0.0072 | *GRB10, CAV1, SOGA1, MSTN, STAT1, PARP1* | 4.9124 | 1.0000 |
| GOTERM_BP_ALL | GO:0032868~response to insulin | 0.0103 | *GRB10, SOGA1, MSTN, STAT1, PARP1* | 5.8228 | 1.0000 |
| GOTERM_CC_DIRECT | GO:0000139~Golgi membrane | 0.0107 | *ICA1, CAV1, HACE1, GALNT11, ARFGEF1* | 5.7801 | 0.7238 |
| GOTERM_BP_ALL | GO:0043067~regulation of programmed cell death | 0.0112 | *CAV1, MCL1, GRIK2, ADAMTSL4, MITF, MSTN, KIT, STAT1, NAA35, DAPK1, OSR1, CTSC, YAP1, CLEC5A* | 2.1541 | 1.0000 |
| GOTERM_CC_ALL | GO:0000139~Golgi membrane | 0.0119 | *ICA1, CAV1, MYO1B, HACE1, GALNT11, ARFGEF1* | 4.3492 | 0.9645 |
| KEGG_PATHWAY | ecb00052:Galactose metabolism | 0.0137 | *MGAM, PGM1, MGAM2* | 16.3072 | 0.7748 |
| GOTERM_BP_ALL | **GO:2000697~negative regulation of epithelial cell differentiation involved in kidney development** | 0.0146 | *OSR1, STAT1* | 135.0899 | 1.0000 |
| GOTERM_BP_ALL | **GO:0072183~negative regulation of nephron tubule epithelial cell differentiation** | 0.0146 | *OSR1, STAT1* | 135.0899 | 1.0000 |
| UP_KEYWORDS | Activator | 0.0148 | *STAT4, STAT1, STAT2* | 15.9498 | 0.7184 |
| GOTERM_BP_ALL | GO:0001656~metanephros development | 0.0151 | *OSR1, SALL1, YAP1, STAT1* | 7.6107 | 1.0000 |
| KEGG_PATHWAY | ecb00500:Starch and sucrose metabolism | 0.0156 | *MGAM, PGM1, MGAM2* | 15.2551 | 0.8165 |
| GOTERM_BP_ALL | GO:0035850~epithelial cell differentiation involved in kidney development | 0.0177 | *OSR1, YAP1, STAT1* | 14.4739 | 1.0000 |
| GOTERM_BP_ALL | GO:0010941~regulation of cell death | 0.0179 | *CAV1, MCL1, GRIK2, ADAMTSL4, MITF, MSTN, KIT, STAT1, NAA35, DAPK1, OSR1, CTSC, YAP1, CLEC5A* | 2.0249 | 1.0000 |
| GOTERM_BP_ALL | GO:0032870~cellular response to hormone stimulus | 0.0180 | *GRB10, CAV1, SOGA1, MSTN, KIT, STAT1, PARP1* | 3.3180 | 1.0000 |
| GOTERM_BP_ALL | **GO:0072162~metanephric mesenchymal cell differentiation** | 0.0218 | *OSR1, STAT1* | 90.0599 | 1.0000 |
| GOTERM_BP_DIRECT | **GO:0072162~metanephric mesenchymal cell differentiation** | 0.0218 | *OSR1, STAT1* | 90.0599 | 1.0000 |
| GOTERM_BP_ALL | GO:0030856~regulation of epithelial cell differentiation | 0.0222 | *CAV1, OSR1, YAP1, STAT1* | 6.5898 | 1.0000 |
| GOTERM_BP_ALL | GO:0042981~regulation of apoptotic process | 0.0240 | *CAV1, MCL1, GRIK2, ADAMTSL4, MITF, MSTN, STAT1, NAA35, DAPK1, OSR1, CTSC, YAP1, CLEC5A* | 2.0209 | 1.0000 |
| GOTERM_BP_ALL | GO:0010463~mesenchymal cell proliferation | 0.0255 | *OSR1, PHF14, STAT1* | 11.9197 | 1.0000 |
| GOTERM_BP_ALL | GO:0090183~regulation of kidney development | 0.0255 | *OSR1, YAP1, STAT1* | 11.9197 | 1.0000 |
| GOTERM_BP_ALL | GO:0071417~cellular response to organonitrogen compound | 0.0258 | *GRB10, CAV1, SOGA1, MSTN, STAT1, PARP1* | 3.5550 | 1.0000 |
| GOTERM_BP_ALL | GO:0072009~nephron epithelium development | 0.0274 | *OSR1, SALL1, YAP1, STAT1* | 6.0715 | 1.0000 |
| GOTERM_BP_ALL | GO:0043903~regulation of symbiosis, encompassing mutualism through parasitism | 0.0285 | *CAV1, IL23A, FAM111A, MSTN, STAT1, STAT2* | 3.4638 | 1.0000 |
| GOTERM_CC_DIRECT | GO:0005901~caveola | 0.0289 | *CAV1, KIF18A, MYOF* | 11.1749 | 0.9703 |
| INTERPRO | **IPR020350:Chemokine-like protein, FAM19A2** | 0.0298 | *FAM19A1, FAM19A4* | 65.6903 | 1.0000 |
| GOTERM_BP_ALL | GO:0061005~cell differentiation involved in kidney development | 0.0299 | *OSR1, YAP1, STAT1* | 10.9532 | 1.0000 |
| GOTERM_MF_ALL | GO:0008235~metalloexopeptidase activity | 0.0307 | *CPA6, AGTPBP1, XPNPEP1* | 10.7795 | 1.0000 |
| INTERPRO | IPR008967:p53-like transcription factor, DNA-binding | 0.0332 | *STAT4, STAT1, STAT2* | 10.3721 | 1.0000 |
| GOTERM_BP_ALL | GO:0042509~regulation of tyrosine phosphorylation of STAT protein | 0.0345 | *CAV1, IL23A, KIT* | 10.1317 | 1.0000 |
| GOTERM_CC_ALL | GO:0098588~bounding membrane of organelle | 0.0345 | *MTMR2, PEX11B, ICA1, CAV1, MCL1, SYNDIG1, MYO1B, HACE1, GALNT11, PEX5L, ARFGEF1* | 2.0983 | 0.9999 |
| GOTERM_CC_ALL | GO:0044431~Golgi apparatus part | 0.0354 | *SNX9, ICA1, CAV1, MYO1B, HACE1, GALNT11, ARFGEF1* | 2.8445 | 1.0000 |
| GOTERM_MF_DIRECT | GO:0004871~signal transducer activity | 0.0355 | *STAT4, STAT1, ECM1, STAT2* | 5.4693 | 0.9886 |
| GOTERM_BP_ALL | **GO:0072307~regulation of metanephric nephron tubule epithelial cell differentiation** | 0.0361 | *YAP1, STAT1* | 54.0360 | 1.0000 |
| GOTERM_BP_DIRECT | **GO:0001960~negative regulation of cytokine-mediated signaling pathway** | 0.0361 | *CAV1, ECM1* | 54.0360 | 1.0000 |
| GOTERM_BP_DIRECT | **GO:0030857~negative regulation of epithelial cell differentiation** | 0.0361 | *CAV1, YAP1* | 54.0360 | 1.0000 |
| GOTERM_BP_ALL | GO:0007169~transmembrane receptor protein tyrosine kinase signaling pathway | 0.0365 | *GRB10, SOGA1, PHF14, ROR1, MSTN, KIT, CLNK* | 2.8144 | 1.0000 |
| INTERPRO | IPR003598:Immunoglobulin subtype 2 | 0.0370 | *MDGA2, ROR1, LRIG3, KIT, LRFN5* | 3.9572 | 1.0000 |
| INTERPRO | IPR006581:VPS10 | 0.0372 | *SORCS1, SORCS2* | 52.5522 | 1.0000 |
| GOTERM_BP_ALL | GO:0044763~single-organism cellular process | 0.0379 | *TARS2, AGTPBP1, GRIK2, PRKAG2, MITF, CLNK, GALNTL5, OSR1, NPSR1, YAP1, USP13, RBFOX1, DDC, SOGA1, TTC7A, RBL1, DAPK1, GRM5, GRB10, TIMELESS, FRMD4B, NAB1, ROR1, CTSC, CLEC5A, INPP1, SNX9, CAV1, SYNDIG1, MCL1, CEP126, FRYL, BBS9, ADAMTSL4, ITGA10, KIT, CEP55, PEX5L, ARFGEF1, NAA35, ALCAM, MIP, MTMR2, STAT4, IL23A, FBXO43, EXOC6, GALNT11, MYOF, FBXW11, AFAP1, ICA1, MYO1B, KIF18A, HACE1, SAMHD1, MSTN, STAT1, AMPD3, ECM1, STAT2, CSPP1, PEX11B, PHF14, SLC16A7, ETS1, SALL1, WDR1, HIBCH, PARP1* | 1.1345 | 1.0000 |
| GOTERM_BP_ALL | GO:0009725~response to hormone | 0.0383 | *GRB10, CAV1, SOGA1, MSTN, KIT, STAT1, PARP1* | 2.7813 | 1.0000 |
| GOTERM_BP_ALL | GO:0012501~programmed cell death | 0.0401 | *CAV1, MCL1, GRIK2, ADAMTSL4, MITF, MSTN, KIT, STAT1, NAA35, DAPK1, OSR1, CTSC, YAP1, CLEC5A* | 1.8098 | 1.0000 |
| GOTERM_BP_ALL | GO:0035295~tube development | 0.0408 | *OSR1, TIMELESS, PHF14, SALL1, KIF18A, YAP1, KIT, STAT1* | 2.4730 | 1.0000 |
| GOTERM_BP_ALL | GO:0072006~nephron development | 0.0416 | *OSR1, SALL1, YAP1, STAT1* | 5.1463 | 1.0000 |
| SMART | **SM00602:VPS10** | 0.0418 | *SORCS1, SORCS2* | 46.3818 | 0.9776 |
| GOTERM_CC_ALL | GO:0005901~caveola | 0.0418 | *CAV1, KIF18A, MYOF* | 9.1431 | 1.0000 |
| GOTERM_BP_ALL | GO:0043069~negative regulation of programmed cell death | 0.0418 | *CAV1, OSR1, MCL1, GRIK2, YAP1, KIT, CLEC5A, NAA35, DAPK1* | 2.2725 | 1.0000 |
| GOTERM_BP_ALL | **GO:0072161~mesenchymal cell differentiation involved in kidney development** | 0.0431 | *OSR1, STAT1* | 45.0300 | 1.0000 |
| GOTERM_BP_ALL | **GO:2001012~mesenchymal cell differentiation involved in renal system development** | 0.0431 | *OSR1, STAT1* | 45.0300 | 1.0000 |
| GOTERM_BP_ALL | **GO:0072203~cell proliferation involved in metanephros development** | 0.0431 | *OSR1, STAT1* | 45.0300 | 1.0000 |
| GOTERM_BP_DIRECT | **GO:0019217~regulation of fatty acid metabolic process** | 0.0431 | *CAV1, PRKAG2* | 45.0300 | 1.0000 |
| GOTERM_CC_ALL | GO:0099572~postsynaptic specialization | 0.0435 | *GRM5, SYNDIG1, GRIK2* | 8.9399 | 1.0000 |
| GOTERM_CC_ALL | GO:0014069~postsynaptic density | 0.0435 | *GRM5, SYNDIG1, GRIK2* | 8.9399 | 1.0000 |
| GOTERM_CC_DIRECT | GO:0014069~postsynaptic density | 0.0435 | *GRM5, SYNDIG1, GRIK2* | 8.9399 | 0.9952 |
| GOTERM_BP_ALL | GO:0007260~tyrosine phosphorylation of STAT protein | 0.0445 | *CAV1, IL23A, KIT* | 8.8102 | 1.0000 |
| GOTERM_BP_ALL | GO:0048513~animal organ development | 0.0458 | *RBFOX1, CAV1, TTC7A, AGTPBP1, MITF, KIF18A, MSTN, KIT, LRIG3, STAT1, ECM1, PCDH18, MIP, IL23A, OSR1, PHF14, TIMELESS, ETS1, HOXC4, SALL1, NAB1, YAP1, CLEC5A* | 1.4824 | 1.0000 |
| GOTERM_BP_ALL | GO:0006952~defense response | 0.0460 | *CAV1, IL23A, GRIK2, ETS1, MSTN, SAMHD1, KIT, STAT1, CLEC5A, ECM1, STAT2, DAPK1* | 1.9027 | 1.0000 |
| GOTERM_BP_ALL | GO:0006357~regulation of transcription from RNA polymerase II promoter | 0.0467 | *CAV1, LCORL, RBL1, MITF, STAT1, ECM1, STAT4, IL23A, OSR1, PHF14, TIMELESS, ETS1, SALL1, YAP1* | 1.7692 | 1.0000 |
| GOTERM_BP_ALL | GO:0034097~response to cytokine | 0.0472 | *STAT4, CAV1, MCL1, KIT, STAT1, ECM1, DAPK1* | 2.6414 | 1.0000 |
| GOTERM_BP_ALL | GO:0051259~protein oligomerization | 0.0472 | *MIP, MTMR2, PEX11B, SNX9, CAV1, SAMHD1* | 3.0132 | 1.0000 |
| GOTERM_BP_ALL | GO:1901699~cellular response to nitrogen compound | 0.0472 | *GRB10, CAV1, SOGA1, MSTN, STAT1, PARP1* | 3.0132 | 1.0000 |
| GOTERM_CC_ALL | GO:0098805~whole membrane | 0.0482 | *MTMR2, PEX11B, ICA1, CAV1, MCL1, SYNDIG1, MYO1B, KIF18A, PEX5L, MYOF* | 2.0823 | 1.0000 |
| GOTERM_BP_ALL | GO:0072073~kidney epithelium development | 0.0499 | *OSR1, SALL1, YAP1, STAT1* | 4.7819 | 1.0000 |
| INTERPRO | IPR003599:Immunoglobulin subtype | 0.0503 | *ALCAM, MDGA2, ROR1, LRIG3, KIT, LRFN5* | 2.9747 | 1.0000 |
| GOTERM_BP_ALL | GO:0007166~cell surface receptor signaling pathway | 0.0518 | *CAV1, SOGA1, MCL1, GRIK2, MSTN, ITGA10, KIT, CLNK, STAT1, ECM1, DAPK1, GRM5, STAT4, GRB10, PHF14, SALL1, ROR1, YAP1* | 1.5831 | 1.0000 |
| SMART | SM00408:IGc2 | 0.0521 | *MDGA2, ROR1, LRIG3, KIT, LRFN5* | 3.4926 | 0.9914 |
| KEGG_PATHWAY | ecb04630:Jak-STAT signaling pathway | 0.0524 | *STAT4, IL23A, STAT1, STAT2* | 4.6025 | 0.9970 |
| GOTERM_BP_ALL | GO:0010648~negative regulation of cell communication | 0.0527 | *GRB10, CAV1, MCL1, PHF14, GRIK2, MSTN, YAP1, STAT1, FBXW11, ECM1, DAPK1* | 1.9399 | 1.0000 |
| GOTERM_BP_ALL | GO:2001236~regulation of extrinsic apoptotic signaling pathway | 0.0532 | *CAV1, MCL1, YAP1, DAPK1* | 4.6583 | 1.0000 |
| GOTERM_BP_ALL | GO:0023057~negative regulation of signaling | 0.0539 | *GRB10, CAV1, MCL1, PHF14, GRIK2, MSTN, YAP1, STAT1, FBXW11, ECM1, DAPK1* | 1.9324 | 1.0000 |
| GOTERM_CC_ALL | GO:0060076~excitatory synapse | 0.0545 | *GRM5, SYNDIG1, GRIK2* | 7.8882 | 1.0000 |
| GOTERM_CC_ALL | GO:0044853~plasma membrane raft | 0.0545 | *CAV1, KIF18A, MYOF* | 7.8882 | 1.0000 |
| GOTERM_BP_ALL | GO:0050678~regulation of epithelial cell proliferation | 0.0555 | *CAV1, OSR1, YAP1, STAT1, ECM1* | 3.4462 | 1.0000 |
| KEGG_PATHWAY | ecb05321:Inflammatory bowel disease (IBD) | 0.0562 | *STAT4, IL23A, STAT1* | 7.6276 | 0.9981 |
| INTERPRO | IPR013098:Immunoglobulin I-set | 0.0562 | *MDGA2, ROR1, LRIG3, LRFN5* | 4.5698 | 1.0000 |
| GOTERM_BP_ALL | GO:1901701~cellular response to oxygen-containing compound | 0.0563 | *GRB10, CAV1, SOGA1, OSR1, MSTN, YAP1, STAT1, PARP1* | 2.2994 | 1.0000 |
| GOTERM_BP_ALL | GO:0007259~JAK-STAT cascade | 0.0566 | *CAV1, IL23A, KIT, STAT1* | 4.5408 | 1.0000 |
| GOTERM_BP_ALL | **GO:0051103~DNA ligation involved in DNA repair** | 0.0571 | *XRCC4, PARP1* | 33.7725 | 1.0000 |
| GOTERM_BP_DIRECT | **GO:0008406~gonad development** | 0.0571 | *OSR1, SALL1* | 33.7725 | 1.0000 |
| GOTERM_BP_DIRECT | **GO:0051103~DNA ligation involved in DNA repair** | 0.0571 | *XRCC4, PARP1* | 33.7725 | 1.0000 |
| GOTERM_BP_ALL | GO:0006366~transcription from RNA polymerase II promoter | 0.0581 | *CAV1, LCORL, MITF, RBL1, STAT1, STAT4, IL23A, OSR1, PHF14, TIMELESS, ETS1, SALL1, YAP1* | 1.7650 | 1.0000 |
| GOTERM_BP_ALL | GO:0060548~negative regulation of cell death | 0.0587 | *CAV1, OSR1, MCL1, GRIK2, YAP1, KIT, CLEC5A, NAA35, DAPK1* | 2.1181 | 1.0000 |
| GOTERM_CC_ALL | GO:0044421~extracellular region part | 0.0596 | *SNX9, ADAMTSL4, PRKAG2, KIT, LRIG3, CEP55, MTMR2, ALCAM, IL23A, RPS29, MYOF, GOLM1, NDUFA4, DDC, SOGA1, CPA6, MYO1B, MSTN, ISOC1, ECM1, XPNPEP1, GRM5, AMBP, PEX11B, FRMD4B, MGAM, CTSC, HIBCH, WDR1* | 1.3640 | 1.0000 |
| GOTERM_BP_ALL | GO:0044699~single-organism process | 0.0596 | *TARS2, AGTPBP1, GRIK2, PRKAG2, MITF, CLNK, GALNTL5, OSR1, HOXC4, NPSR1, YAP1, USP13, RBFOX1, DDC, SOGA1, TTC7A, RBL1, DAPK1, GRM5, GRB10, TIMELESS, ASCC3, FRMD4B, NAB1, ROR1, SLC27A6, CTSC, CLEC5A, INPP1, SNX9, CAV1, SYNDIG1, MCL1, FRYL, CEP126, ADAMTSL4, BBS9, ITGA10, KIT, CEP55, LRIG3, PEX5L, ARFGEF1, NAA35, ALCAM, MIP, MTMR2, STAT4, IL23A, FBXO43, EXOC6, GALNT11, MYOF, FBXW11, AFAP1, ICA1, MYO1B, KIF18A, HACE1, SAMHD1, MSTN, STAT1, AMPD3, ECM1, STAT2, PCDH18, CSPP1, PEX11B, PHF14, SLC16A7, ETS1, SALL1, HIBCH, WDR1, PARP1* | 1.0989 | 1.0000 |
| GOTERM_BP_ALL | GO:0097696~STAT cascade | 0.0601 | *CAV1, IL23A, KIT, STAT1* | 4.4292 | 1.0000 |
| GOTERM_BP_ALL | GO:0008406~gonad development | 0.0601 | *OSR1, SALL1, KIF18A, KIT* | 4.4292 | 1.0000 |
| GOTERM_CC_DIRECT | GO:0005938~cell cortex | 0.0603 | *CAV1, FRYL, ADD3* | 7.4499 | 0.9994 |
| GOTERM_BP_ALL | GO:0008219~cell death | 0.0605 | *CAV1, MCL1, GRIK2, ADAMTSL4, MITF, MSTN, KIT, STAT1, NAA35, DAPK1, OSR1, CTSC, YAP1, CLEC5A* | 1.7008 | 1.0000 |
| GOTERM_BP_DIRECT | GO:0045944~positive regulation of transcription from RNA polymerase II promoter | 0.0606 | *STAT4, IL23A, OSR1, ETS1, SALL1, MITF, YAP1, STAT1* | 2.2609 | 1.0000 |
| GOTERM_BP_ALL | GO:0045137~development of primary sexual characteristics | 0.0637 | *OSR1, SALL1, KIF18A, KIT* | 4.3229 | 1.0000 |
| GOTERM_BP_ALL | **GO:0072202~cell differentiation involved in metanephros development** | 0.0640 | *OSR1, STAT1* | 30.0200 | 1.0000 |
| GOTERM_BP_ALL | **GO:0046855~inositol phosphate dephosphorylation** | 0.0640 | *INPP1, MTMR2* | 30.0200 | 1.0000 |
| GOTERM_BP_ALL | **GO:0097531~mast cell migration** | 0.0640 | *MITF, KIT* | 30.0200 | 1.0000 |
| GOTERM_BP_ALL | **GO:0072075~metanephric mesenchyme development** | 0.0640 | *OSR1, STAT1* | 30.0200 | 1.0000 |
| GOTERM_BP_ALL | **GO:0046838~phosphorylated carbohydrate dephosphorylation** | 0.0640 | *INPP1, MTMR2* | 30.0200 | 1.0000 |
| GOTERM_BP_ALL | **GO:0071545~inositol phosphate catabolic process** | 0.0640 | *INPP1, MTMR2* | 30.0200 | 1.0000 |
| GOTERM_BP_DIRECT | **GO:0046855~inositol phosphate dephosphorylation** | 0.0640 | *INPP1, MTMR2* | 30.0200 | 1.0000 |
| UP_KEYWORDS | Phosphoprotein | 0.0644 | *STAT4, CAV1, STAT1, STAT2* | 4.3224 | 0.9965 |
| GOTERM_BP_ALL | GO:0016032~viral process | 0.0673 | *CAV1, IL23A, FAM111A, MSTN, SAMHD1, STAT1, STAT2* | 2.4123 | 1.0000 |
| GOTERM_BP_ALL | GO:0043900~regulation of multi-organism process | 0.0681 | *CAV1, IL23A, FAM111A, MSTN, STAT1, STAT2* | 2.7108 | 1.0000 |
| GOTERM_MF_ALL | GO:0008237~metallopeptidase activity | 0.0686 | *CPA6, AGTPBP1, ADAMTSL4, XPNPEP1* | 4.1759 | 1.0000 |
| GOTERM_BP_ALL | GO:0044764~multi-organism cellular process | 0.0687 | *CAV1, IL23A, FAM111A, MSTN, SAMHD1, STAT1, STAT2* | 2.4001 | 1.0000 |
| GOTERM_CC_ALL | GO:0005615~extracellular space | 0.0697 | *AMBP, CPA6, SOGA1, IL23A, FRMD4B, PRKAG2, MSTN, CTSC, LRIG3, KIT, ECM1, GOLM1* | 1.7781 | 1.0000 |
| GOTERM_BP_ALL | GO:0034645~cellular macromolecule biosynthetic process | 0.0699 | *CAV1, LCORL, TARS2, MITF, KIT, ARFGEF1, STAT4, IL23A, OSR1, RPS29, YAP1, GALNT11, FBXW11, USP13, FAM111A, RBL1, MSTN, STAT1, ECM1, STAT2, DAPK1, ZDHHC14, TIMELESS, PHF14, ETS1, SALL1, NAB1, PARP1* | 1.3461 | 1.0000 |
| GOTERM_BP_ALL | GO:0050792~regulation of viral process | 0.0718 | *IL23A, FAM111A, MSTN, STAT1, STAT2* | 3.1563 | 1.0000 |
| GOTERM_BP_ALL | GO:0006351~transcription, DNA-templated | 0.0721 | *CAV1, LCORL, RBL1, MITF, MSTN, KIT, STAT1, ECM1, STAT2, STAT4, IL23A, OSR1, PHF14, TIMELESS, ETS1, SALL1, NAB1, YAP1, FBXW11* | 1.4905 | 1.0000 |
| GOTERM_BP_ALL | GO:2000026~regulation of multicellular organismal development | 0.0724 | *RBFOX1, CAV1, SYNDIG1, MITF, MSTN, KIT, STAT1, ECM1, MTMR2, IL23A, OSR1, PHF14, ETS1, YAP1* | 1.6532 | 1.0000 |
| GOTERM_BP_ALL | GO:0051093~negative regulation of developmental process | 0.0727 | *MTMR2, CAV1, OSR1, PHF14, MSTN, YAP1, STAT1, ECM1* | 2.1658 | 1.0000 |
| INTERPRO | **IPR000601:PKD domain** | 0.0730 | *SORCS1, SORCS2* | 26.2761 | 1.0000 |
| SMART | SM00409:IG | 0.0738 | *ALCAM, MDGA2, ROR1, LRIG3, KIT, LRFN5* | 2.6254 | 0.9989 |
| GOTERM_BP_ALL | GO:0071345~cellular response to cytokine stimulus | 0.0744 | *STAT4, CAV1, KIT, STAT1, ECM1, DAPK1* | 2.6402 | 1.0000 |
| GOTERM_BP_ALL | GO:0008286~insulin receptor signaling pathway | 0.0755 | *GRB10, SOGA1, MSTN* | 6.5366 | 1.0000 |
| GOTERM_BP_ALL | GO:0019221~cytokine-mediated signaling pathway | 0.0757 | *STAT4, CAV1, KIT, STAT1, ECM1* | 3.0984 | 1.0000 |
| GOTERM_CC_ALL | GO:0031090~organelle membrane | 0.0761 | *NDUFA4, SNX9, NDUFB5, ICA1, CAV1, SYNDIG1, MCL1, MYO1B, HACE1, ARFGEF1, PEX5L, MTMR2, PEX11B, GALNT11* | 1.6468 | 1.0000 |
| GOTERM_BP_DIRECT | GO:0007169~transmembrane receptor protein tyrosine kinase signaling pathway | 0.0777 | *ROR1, KIT, CLNK* | 6.4329 | 1.0000 |
| GOTERM_BP_ALL | **GO:0042523~positive regulation of tyrosine phosphorylation of Stat5 protein** | 0.0777 | *IL23A, KIT* | 24.5618 | 1.0000 |
| GOTERM_BP_DIRECT | **GO:0042523~positive regulation of tyrosine phosphorylation of Stat5 protein** | 0.0777 | *IL23A, KIT* | 24.5618 | 1.0000 |
| GOTERM_BP_ALL | GO:0048568~embryonic organ development | 0.0785 | *OSR1, SALL1, HOXC4, YAP1, LRIG3, KIT* | 2.5979 | 1.0000 |
| GOTERM_MF_ALL | GO:0008238~exopeptidase activity | 0.0791 | *CPA6, AGTPBP1, XPNPEP1* | 6.3496 | 1.0000 |
| GOTERM_BP_ALL | GO:0001936~regulation of endothelial cell proliferation | 0.0798 | *CAV1, STAT1, ECM1* | 6.3323 | 1.0000 |
| GOTERM_BP_ALL | GO:0009968~negative regulation of signal transduction | 0.0816 | *GRB10, CAV1, MCL1, PHF14, MSTN, YAP1, STAT1, FBXW11, ECM1, DAPK1* | 1.8685 | 1.0000 |
| GOTERM_BP_ALL | GO:0048863~stem cell differentiation | 0.0819 | *OSR1, YAP1, KIT* | 6.2349 | 1.0000 |
| GOTERM_BP_ALL | **GO:0072074~kidney mesenchyme development** | 0.0844 | *OSR1, STAT1* | 22.5150 | 1.0000 |
| GOTERM_BP_ALL | **GO:0072234~metanephric nephron tubule development** | 0.0844 | *OSR1, STAT1* | 22.5150 | 1.0000 |
| GOTERM_BP_ALL | **GO:0006266~DNA ligation** | 0.0844 | *XRCC4, PARP1* | 22.5150 | 1.0000 |
| GOTERM_BP_ALL | **GO:0046174~polyol catabolic process** | 0.0844 | *INPP1, MTMR2* | 22.5150 | 1.0000 |
| GOTERM_BP_ALL | **GO:2001014~regulation of skeletal muscle cell differentiation** | 0.0844 | *RBFOX1, MSTN* | 22.5150 | 1.0000 |
| GOTERM_BP_ALL | **GO:0072111~cell proliferation involved in kidney development** | 0.0844 | *OSR1, STAT1* | 22.5150 | 1.0000 |
| GOTERM_BP_ALL | GO:0043065~positive regulation of apoptotic process | 0.0853 | *CAV1, MCL1, ADAMTSL4, CTSC, STAT1, DAPK1* | 2.5329 | 1.0000 |
| GOTERM_BP_ALL | GO:0044419~interspecies interaction between organisms | 0.0864 | *CAV1, IL23A, FAM111A, MSTN, SAMHD1, STAT1, STAT2* | 2.2569 | 1.0000 |
| GOTERM_BP_ALL | GO:0044403~symbiosis, encompassing mutualism through parasitism | 0.0864 | *CAV1, IL23A, FAM111A, MSTN, SAMHD1, STAT1, STAT2* | 2.2569 | 1.0000 |
| GOTERM_BP_ALL | GO:0048562~embryonic organ morphogenesis | 0.0871 | *OSR1, SALL1, HOXC4, YAP1, LRIG3* | 2.9496 | 1.0000 |
| GOTERM_CC_ALL | GO:0005938~cell cortex | 0.0878 | *CAV1, FRYL, EXOC6, ADD3* | 3.7774 | 1.0000 |
| GOTERM_BP_ALL | GO:0043068~positive regulation of programmed cell death | 0.0879 | *CAV1, MCL1, ADAMTSL4, CTSC, STAT1, DAPK1* | 2.5094 | 1.0000 |
| GOTERM_BP_ALL | GO:0001944~vasculature development | 0.0879 | *CAV1, OSR1, ETS1, YAP1, KIT, STAT1, ECM1* | 2.2462 | 1.0000 |
| GOTERM_BP_ALL | GO:0043066~negative regulation of apoptotic process | 0.0895 | *CAV1, OSR1, MCL1, GRIK2, YAP1, CLEC5A, NAA35, DAPK1* | 2.0585 | 1.0000 |
| GOTERM_MF_ALL | GO:0016874~ligase activity | 0.0896 | *TARS2, HACE1, SLC27A6, PARP1* | 3.7231 | 1.0000 |
| GOTERM_BP_ALL | GO:0046434~organophosphate catabolic process | 0.0907 | *INPP1, MTMR2, SAMHD1* | 5.8735 | 1.0000 |
| GOTERM_BP_ALL | **GO:0042510~regulation of tyrosine phosphorylation of Stat1 protein** | 0.0911 | *IL23A, KIT* | 20.7831 | 1.0000 |
| GOTERM_BP_ALL | **GO:0072215~regulation of metanephros development** | 0.0911 | *YAP1, STAT1* | 20.7831 | 1.0000 |
| GOTERM_BP_ALL | **GO:0090185~negative regulation of kidney development** | 0.0911 | *OSR1, STAT1* | 20.7831 | 1.0000 |
| GOTERM_BP_ALL | **GO:0072087~renal vesicle development** | 0.0911 | *OSR1, SALL1* | 20.7831 | 1.0000 |
| GOTERM_BP_ALL | **GO:2000811~negative regulation of anoikis** | 0.0911 | *CAV1, MCL1* | 20.7831 | 1.0000 |
| GOTERM_BP_DIRECT | **GO:2000811~negative regulation of anoikis** | 0.0911 | *CAV1, MCL1* | 20.7831 | 1.0000 |
| GOTERM_BP_DIRECT | **GO:0048169~regulation of long-term neuronal synaptic plasticity** | 0.0911 | *GRM5, GRIK2* | 20.7831 | 1.0000 |
| GOTERM_BP_ALL | GO:0009059~macromolecule biosynthetic process | 0.0926 | *CAV1, LCORL, TARS2, MITF, KIT, ARFGEF1, STAT4, IL23A, OSR1, RPS29, YAP1, GALNT11, FBXW11, USP13, FAM111A, RBL1, MSTN, STAT1, ECM1, STAT2, DAPK1, ZDHHC14, TIMELESS, PHF14, ETS1, SALL1, NAB1, PARP1* | 1.3088 | 1.0000 |
| GOTERM_BP_ALL | GO:2001237~negative regulation of extrinsic apoptotic signaling pathway | 0.0930 | *MCL1, YAP1, DAPK1* | 5.7896 | 1.0000 |
| GOTERM_MF_ALL | GO:0008270~zinc ion binding | 0.0930 | *DTX4, ZDHHC14, CPA6, RPS29, PHF14, AGTPBP1, ADAMTSL4, SAMHD1, PARP1, USP13* | 1.8071 | 1.0000 |
| GOTERM_MF_DIRECT | GO:0008270~zinc ion binding | 0.0930 | *DTX4, ZDHHC14, CPA6, RPS29, PHF14, AGTPBP1, ADAMTSL4, SAMHD1, PARP1, USP13* | 1.8071 | 1.0000 |
| GOTERM_BP_ALL | GO:0050673~epithelial cell proliferation | 0.0936 | *CAV1, OSR1, YAP1, STAT1, ECM1* | 2.8743 | 1.0000 |
| GOTERM_CC_DIRECT | GO:0005667~transcription factor complex | 0.0950 | *ETS1, RBL1, YAP1, PARP1* | 3.6490 | 1.0000 |
| GOTERM_BP_ALL | GO:0010243~response to organonitrogen compound | 0.0952 | *GRB10, CAV1, SOGA1, MSTN, STAT1, PARP1* | 2.4488 | 1.0000 |
| GOTERM_BP_ALL | GO:0010942~positive regulation of cell death | 0.0952 | *CAV1, MCL1, ADAMTSL4, CTSC, STAT1, DAPK1* | 2.4488 | 1.0000 |
| GOTERM_BP_ALL | GO:0006955~immune response | 0.0959 | *CAV1, IL23A, SAMHD1, CTSC, KIT, CLNK, STAT1, CLEC5A, ECM1, DAPK1* | 1.8060 | 1.0000 |
| GOTERM_BP_ALL | GO:1901342~regulation of vasculature development | 0.0973 | *ETS1, KIT, STAT1, ECM1* | 3.6024 | 1.0000 |
| GOTERM_MF_ALL | GO:0004181~metallocarboxypeptidase activity | 0.0975 | *CPA6, AGTPBP1* | 19.3134 | 1.0000 |
| GOTERM_MF_DIRECT | GO:0004181~metallocarboxypeptidase activity | 0.0975 | *CPA6, AGTPBP1* | 19.3134 | 1.0000 |
| GOTERM_BP_ALL | GO:0072170~metanephric tubule development | 0.0978 | *OSR1, STAT1* | 19.2986 | 1.0000 |
| GOTERM_BP_ALL | GO:0006950~response to stress | 0.0996 | *XRCC4, CAV1, MCL1, GRIK2, SAMHD1, MSTN, KIT, STAT1, SMUG1, ECM1, ARFGEF1, STAT2, DAPK1, IL23A, TIMELESS, ETS1, ASCC3, YAP1, PARP1, MYOF, CLEC5A, USP13* | 1.3740 | 1.0000 |

Figure S2. Six first functional annotation clusters with cluster enrichment values above or close to 1. Green colour marks for corresponding gene-term association to have been positively reported, grey indicates no reported gene-term association thus far.


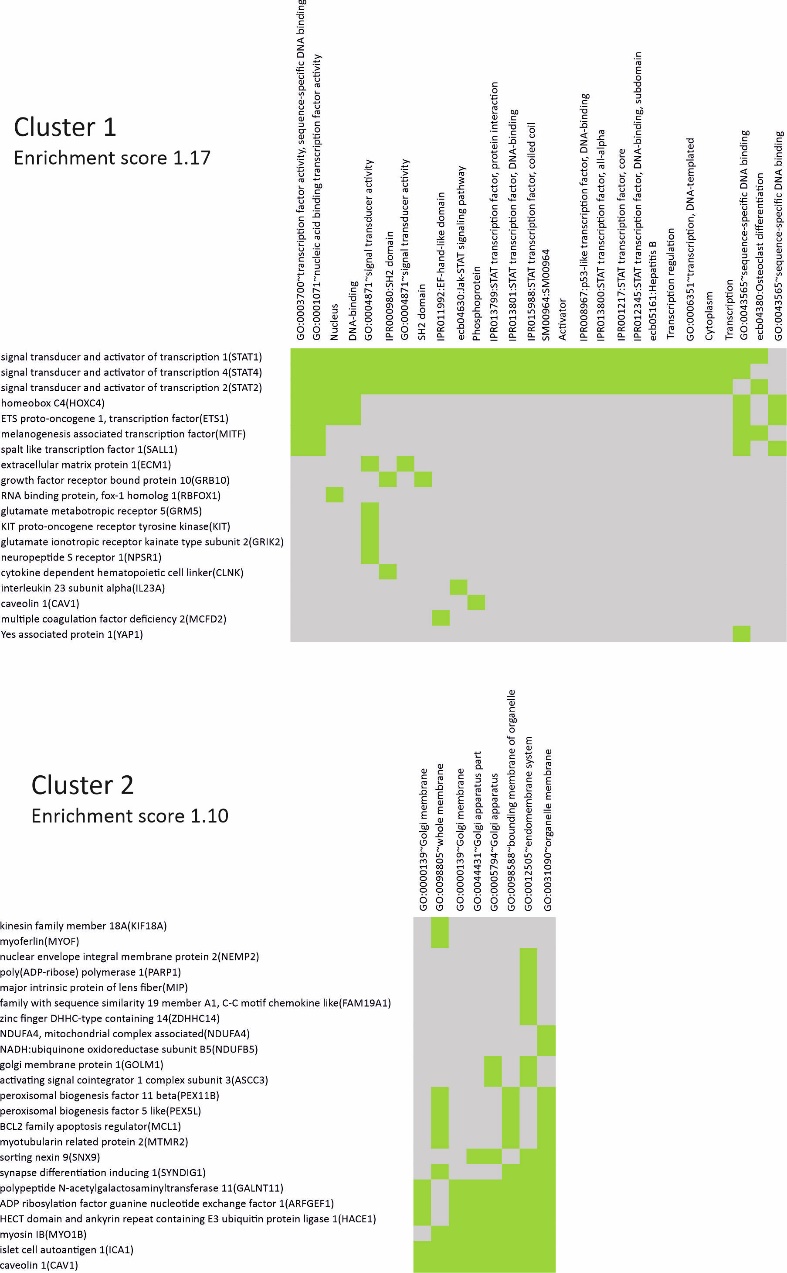

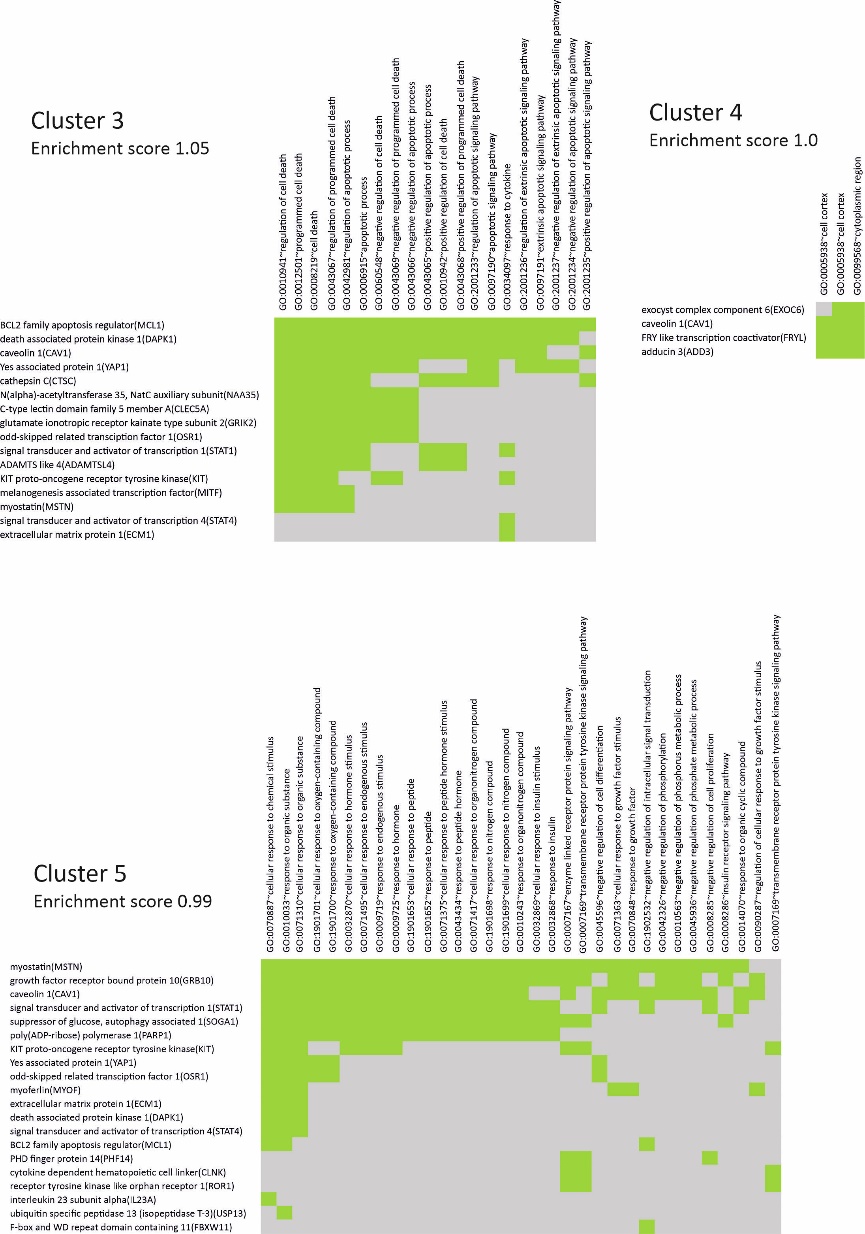

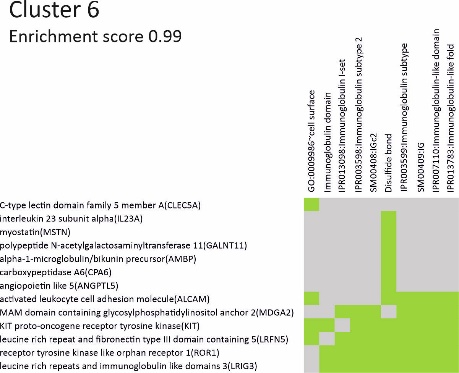

Supplement: Supplementary file 1 — Supplementary Material [file JBG-138-188-s001.docx]
